# Reprogramming of microRNA expression via E2F1 downregulation promotes *Salmonella* infection both in infected and bystander cells

**DOI:** 10.1038/s41467-021-23593-z

**Published:** 2021-06-07

**Authors:** Carmen Aguilar, Susana Costa, Claire Maudet, R. P. Vivek-Ananth, Sara Zaldívar-López, Juan J. Garrido, Areejit Samal, Miguel Mano, Ana Eulalio

**Affiliations:** 1 Host RNA Metabolism Group, Institute for Molecular Infection Biology (IMIB), University of Würzburg, Würzburg, Germany; 2 RNA & Infection Laboratory, Center for Neuroscience and Cell Biology (CNC), University of Coimbra, Coimbra, Portugal; 3 Functional Genomics and RNA-based Therapeutics Laboratory, Center for Neuroscience and Cell Biology (CNC), University of Coimbra, Coimbra, Portugal; 4 PhD Programme in Experimental Biology and Biomedicine (PDBEB), Institute for Interdisciplinary Research (IIIUC), University of Coimbra, Coimbra, Portugal; 5 The Institute of Mathematical Sciences (IMSc), Homi Bhabha National Institute (HBNI), Chennai, India; 6 Animal Breeding and Genomics Group, Department of Genetics, Faculty of Veterinary Science, University of Córdoba, Córdoba, Spain; 7 Immunogenomics and Molecular Pathogenesis GA14 Group, Maimónides Biomedical Research Institute of Córdoba (IMIBIC), Córdoba, Spain; 8 Department of Life Sciences, University of Coimbra, Coimbra, Portugal; 9 Present Address: Biology of Infection Unit, Institut Pasteur, Paris, France

**Keywords:** Bacterial pathogenesis, Cellular microbiology, Pathogens, miRNAs

## Abstract

Cells infected with pathogens can contribute to clearing infections by releasing signals that instruct neighbouring cells to mount a pro-inflammatory cytokine response, or by other mechanisms that reduce bystander cells’ susceptibility to infection. Here, we show the opposite effect: epithelial cells infected with *Salmonella* Typhimurium secrete host factors that facilitate the infection of bystander cells. We find that the endoplasmic reticulum stress response is activated in both infected and bystander cells, and this leads to activation of JNK pathway, downregulation of transcription factor E2F1, and consequent reprogramming of microRNA expression in a time-dependent manner. These changes are not elicited by infection with other bacterial pathogens, such as *Shigella flexneri* or *Listeria monocytogenes*. Remarkably, the protein HMGB1 present in the secretome of *Salmonella*-infected cells is responsible for the activation of the IRE1 branch of the endoplasmic reticulum stress response in non-infected, neighbouring cells. Furthermore, E2F1 downregulation and the associated microRNA alterations promote *Salmonella* replication within infected cells and prime bystander cells for more efficient infection.

## Abstract (web summary)

Cells infected with pathogens can release signals that instruct neighbouring cells to mount an immune response or that reduce these cells’ susceptibility to infection. Here, Aguilar et al. show the opposite effect: cells infected with *Salmonella* Typhimurium secrete host factors that facilitate the infection of bystander cells by activating their ER-stress response.

## Introduction

MicroRNAs (miRNAs), due to their instrumental role as post-transcriptional regulators of gene expression^1^, are increasingly recognized as major players in the interaction between host cells and bacterial pathogens^2^. Although miRNAs were initially shown to be part of the host immune response to fight infection, emerging evidence demonstrates that the host miRNA pathway can also be subverted by bacterial pathogens for their own benefit.

Advances in RNA sequencing have contributed to revealing that infections by various bacterial pathogens induce extensive changes of the host miRNome. Along this line, it has also been shown that even closely related bacterial species can lead to distinct host miRNome changes^3^ (e.g. virulent vs. avirulent *Mycobacterium tuberculosis*^4^ or *Francisella tularensis* subspecies *tularensis* vs. subspecies *novicida*^5^). Conversely, a common set of miRNAs was shown to be consistently regulated upon infection with six bacteria^6^. Notwithstanding, the molecular mechanisms underlying most of the described miRNA regulations remain poorly understood. Furthermore, the comparative analysis of miRNA profiles in cells with internalized bacterial and neighboring non-infected bystander cells, as well as the evaluation of their potential relevance for infection, has yet to be investigated.

It is becoming increasingly clear that cells infected with pathogens can signal to bystander cells. In this context, cumulating evidence shows that infected cells have the ability to alert and instruct bystander cells to mount a pro-inflammatory cytokine response, thus contributing to clearing infections more effectively. The process of bystander cell activation is indeed emerging as a crucial evolutionary adaptation in metazoan innate immunity, relevant to viral, parasite, and bacterial infections, in a broad range of hosts^7–9^. Although the relevance of bystander innate immune responses has been mostly described for viral infections, bystander cell activation by bacterial pathogens has also been described. A seminal report by Kasper et al.^10^ revealed that infection by *Shigella flexneri* activates the production of the neutrophil chemotactic factor interleukin-8 (IL-8) in bystander cells, whereas its expression is impaired in infected host epithelial cells. IL-8 production is activated through a mitogen-activated protein kinase (MAPK)-dependent mechanism in response to a yet unidentified molecular signal transmitted to adjacent cells via gap junctions. Increased IL-8 production in bystander cells has also been observed in response to infection by the bacterial pathogens *Salmonella* Typhimurium and *Listeria monocytogenes*^10^. Paracrine bystander cell activation mediated by soluble signals has also been reported in response to bacterial pathogen infection. An interesting study revealed that during *Listeria* infection the chemokines CXCL2 and CXCL5 are primarily produced by epithelial bystander cells, rather than by infected cells where their expression is dampened^11^. In this case, reactive oxygen intermediates mediate the paracrine activation of bystander cells. Similarly, during *Chlamydia trachomatis* infection the response of infected cells to interferon-γ is blocked, whereas that of bystander cells is unhindered contributing to limiting the spread of infection^12^. Along the same line, we have shown that *Shigella flexneri* infection induces plasma membrane remodeling in infected and bystander cells, through the activation of the acid sphingomyelinase and strong accumulation of ceramide at the cell surface^13^. These changes of membrane composition determine a depletion of permissive bacterial binding sites, constituting a cell-autonomous defense mechanism that protects cells from infection by non-motile bacteria.

As described above, the study of bystander cells in the context of infection by bacterial pathogens has mainly focused on their role in host defense mechanisms, particularly innate immunity. However, given the constant evolutionary pressure of host and pathogen to obtain a competitive advantage, it is conceivable that both have evolved alternative strategies to exploit bystander cell functions.

Here, we analyzed the role of the transcription factor E2F1 in the regulation of miRNAs upon infection with the bacterial pathogen *Salmonella* *enterica* serovar Typhimurium (hereafter, *Salmonella*). E2F1 is a central player in numerous processes, including cell cycle progression^14^, DNA damage response^15^, senescence^16^, apoptosis^17^, and metabolism^18^. Indeed, E2F1 binds to hundreds of promoter regions of genes involved in numerous cellular pathways^19–21^, including miRNA genes^22–26^. We have previously shown that E2F1 expression is decreased upon *Salmonella* infection^27^. We have now determined that E2F1 is a pivotal player in the regulation of the miRNome during *Salmonella* infection, both in infected and bystander cells. Mechanistically, we show that the secretome of *Salmonella*-infected cells is sufficient to induce E2F1 and miRNA regulation in naive cells, through the activation of the endoplasmic reticulum stress response pathway, involving intercellular signaling mediated by secreted HMGB1 and its interaction with the transmembrane receptor RAGE (receptor for advanced glycation end products). Collectively, this work reveals that the downregulation of E2F1 and consequently of miRNAs promotes infection, by promoting on one hand the bacterial replication within infected cells as well as by priming bystander cells for efficient *Salmonella* infection.

## Results

### E2F1 transcription factor is a major player in miRNA regulation during *Salmonella* infection

**Fig. 1:**
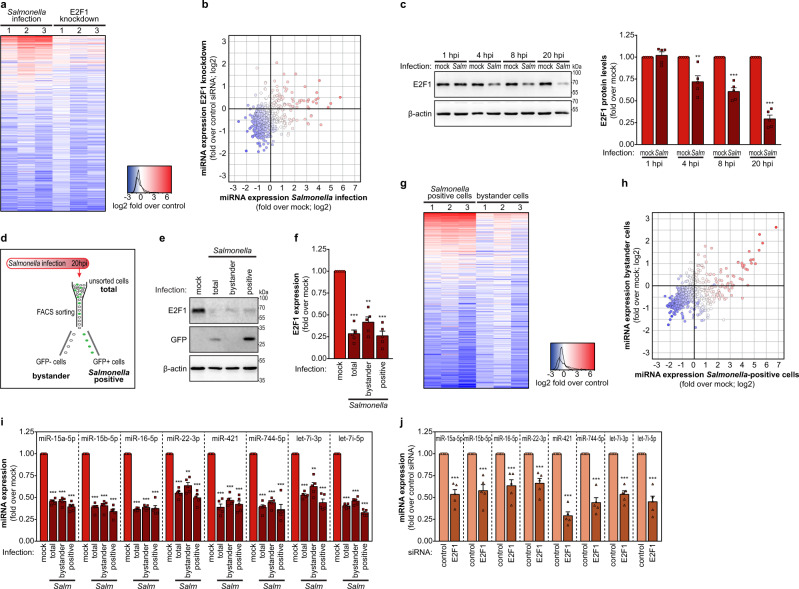
E2F1 plays a crucial role in miRNA regulation in *Salmonella*-infected and bystander cells. **a** Heat map showing miRNA expression changes in *Salmonella*-infected cells and E2F1 knockdown cells. Results are shown as log2 fold change compared to mock-treated cells, or cells transfected with control siRNA, respectively. RNA-seq datasets from three independent experiments are shown. Only miRNAs with a number of reads ≥20 in both controls are shown. **b** Comparison of miRNA expression changes in *Salmonella*-infected cells and E2F1 knockdown cells. Color code for datapoints identical to panel **a**, based on average values. **c** E2F1 protein levels, determined by western blot, in HeLa cells infected with *Salmonella* or mock treated, analyzed at 1, 4, 8, and 20 hpi. **d** Schematic representation of the experimental design. HeLa cells infected with *Salmonella*-expressing GFP were collected at 20 hpi and subjected to FACS to separate the population of cells with internalized bacteria (*Salmonella* positive) and bystander cells. The total population of cells exposed to *Salmonella* collected at 20 hpi (unsorted) and mock-treated cells were used for comparison. **e**, **f** E2F1 expression, determined by western blot (**e**) or qRT-PCR (**f**), in the different cell populations obtained as described in panel **d** (total, *Salmonella* positive, and bystander). Results are normalized to mock‐treated cells. **g** Heat map showing miRNA expression changes upon *Salmonella* infection of HeLa cells, for the cells with internalized *Salmonella* (*Salmonella* positive) and bystander cells. Results are shown as log2 fold change compared to mock-treated cells. RNA-seq datasets from three independent experiments are shown. Only miRNAs with a number of reads ≥20 in the mock-treated cells are shown. **h** Comparison of miRNA expression changes in *Salmonella-*positive and bystander cells. Color code for datapoints identical to panel **g**, based on average values. **i** Expression levels of the mature forms of selected miRNAs (miR-15a-5p, miR-15b-5p, miR-16-5p, miR-22-3p, miR-421, miR-744-5p, let-7i-3p, and let-7i-5p) determined by qRT-PCR in the total population, *Salmonella*-positive and bystander cells. Results are normalized to mock‐treated cells. **j** Expression levels of the mature forms of selected miRNAs determined by qRT-PCR in HeLa cells transfected with control or E2F1 siRNAs. Results are normalized to cells transfected with control siRNA. *Salmonella* infection was performed at MOI 100. Results are shown as mean ± s.e.m. of *n* = 5 (**c**, **f**, **i**, **j**) biologically independent experiments; western blots are representative of *n* = 3 (**e**) or *n* = 5 (**c**) biologically independent experiments; **P < 0.01 and ***P < 0.001 (statistical analysis is detailed in Supplementary Data 1); Source data are provided as a Source Data file.

To investigate the importance of E2F1 downregulation to the miRNA expression changes occurring during *Salmonella* infection, we have performed a comparative analysis of miRNA expression datasets obtained from small RNA sequencing of *Salmonella*-infected HeLa cells and E2F1 knockdown cells. HeLa cells are an epithelial cell line widely used to study infection by bacterial pathogens, including *Salmonella*. Upon *Salmonella* infection, 258 miRNAs (47% of the total detected miRNAs) were downregulated and 112 miRNAs (20% of total) were upregulated (at least 1.4-fold compared to mock-treated cells; Fig. 1a). In E2F1 knockdown cells, 245 miRNAs (44% of total) were downregulated and 41 miRNAs (7.5% of total) were upregulated (at least 1.4-fold compared to cells transfected with control siRNA; Fig. 1a). Most importantly, the comparison of the miRNA expression profiles revealed a clear trend for miRNAs decreased in E2F1 knockdown cells to also be decreased in *Salmonella*-infected cells (Fig. 1b), with 144 (26% of total; 56% of *Salmonella* downregulated) miRNAs downregulated in both conditions. Of note, only miRNAs with ≥20 reads in both control conditions were considered for the analysis (546 miRNAs). Efficient knockdown of E2F1 was confirmed by western blot (Supplementary Fig. 1a). Overall, these results indicate that the transcription factor E2F1 plays a critical role in the regulation of miRNA expression during *Salmonella* infection.

These results prompted us to further characterize the regulation of E2F1 expression during infection. Firstly, we determined the kinetics of E2F1 modulation upon *Salmonella* infection, in samples collected at early (1 and 4 hpi), intermediate (8 hpi), and late stages (20 hpi) of infection. This analysis revealed that E2F1 protein levels are decreased at 4 hpi, gradually lowering with the progression of infection (8 and 20 hpi; Fig. 1c). E2F1 mRNA levels were also decreased at 4, 8, and 20 hpi (Supplementary Fig. 1b). A similar decrease of E2F1 levels was observed in *Salmonella*-infected HCT-8 cells (Supplementary Fig. 1c, d), a colon epithelial cell line also used as a model for bacterial infection studies.

Additionally, we observed that E2F1 expression remains unchanged in HeLa cells treated with purified *Salmonella* LPS (Supplementary Fig. 1e) or heat-killed *Salmonella* (Supplementary Fig. 1f). Indeed, the decrease of E2F1 was dependent on *Salmonella* internalization, since a *Salmonella* mutant strain defective in invasion (ΔSPI-1 strain) was not able to elicit this phenotype, whereas infection with a strain able to invade but defective in intracellular replication (ΔSPI-2 strain) led to E2F1 downregulation (Supplementary Fig. 1f and ref. ^27^). Finally, we determined that *Shigella flexneri* or *Listeria monocytogenes* infection do not affect E2F1 expression, either at early or late times post-infection (Supplementary Fig. 1g, h). These results demonstrate that the strong regulation of E2F1 expression is not a broad and/or unspecific host cell response to bacterial infection, but it is rather restricted/specific to *Salmonella* infection.

### Regulation of E2F1 and miRNA expression by *Salmonella* infection occurs in infected as well as in bystander cells

Given the strong downregulation of E2F1 expression observed upon *Salmonella* infection (30% of control at 20 hpi), and considering that only about 25–30% of the cells are infected in the experimental conditions analyzed, we hypothesized that E2F1 downregulation could occur not only in infected cells but also in the bystander cell population. Thus, we evaluated E2F1 expression in the fraction of HeLa cells with internalized bacteria (*Salmonella* positive), as well as in bystander cells, separated by fluorescence-activated cell sorting (FACS; Fig. 1d). Interestingly, we observed a strong decrease in E2F1 protein and mRNA levels in both cell populations (Fig. 1e, f). Furthermore, we analyzed miRNome changes in these cell populations—of the 563 detected miRNAs (≥20 reads in mock-treated cells), 186 (i.e. 33%) were commonly downregulated in *Salmonella-*positive and bystander populations (at least 1.4-fold compared to mock-treated cells; Fig. 1g, h).

A panel of eight miRNAs (miR-15a-5p, miR-15b-5p, miR-16-5p, miR-22-3p, miR-421, miR-744-5p, let-7i-3p, and let-7i-5p) was selected for further validation by quantitative reverse transcription PCR (qRT-PCR). The selection of these miRNAs was motivated by the miRNA expression datasets described above, our analysis of miRNA function during *Salmonella* infection^27,28^, as well as on having been previously described as E2F1-dependent miRNAs^22,23,25^. In perfect agreement with E2F1 downregulation (Fig. 1e, f), we observed that the expression of these miRNAs was decreased both in *Salmonella-*positive and bystander cell populations (Fig. 1i). Of note, we confirmed that the expression of miR-15a-5p, miR-15b-5p, miR-16-5p, miR-22-3p, miR-421, miR-744-5p, let-7i-3p, and let-7i-5p is also decreased upon E2F1 knockdown of naive cells (Fig. 1j). In addition, we showed that the levels of these miRNAs are restored to normal levels in cells overexpressing EGFP-E2F1 and then infected with *Salmonella* (Supplementary Fig. 2a, b), providing further evidence of a causal relationship between the levels of these miRNAs and E2F1.

Taken together, these data show that the E2F1 downregulation and the consequent decrease of miRNA expression occur both in cells with internalized *Salmonella*, as well as in the bystander cell population.

### The secretome of *Salmonella*-infected cells is sufficient to elicit E2F1 and miRNA regulation in naive cells

**Fig. 2:**
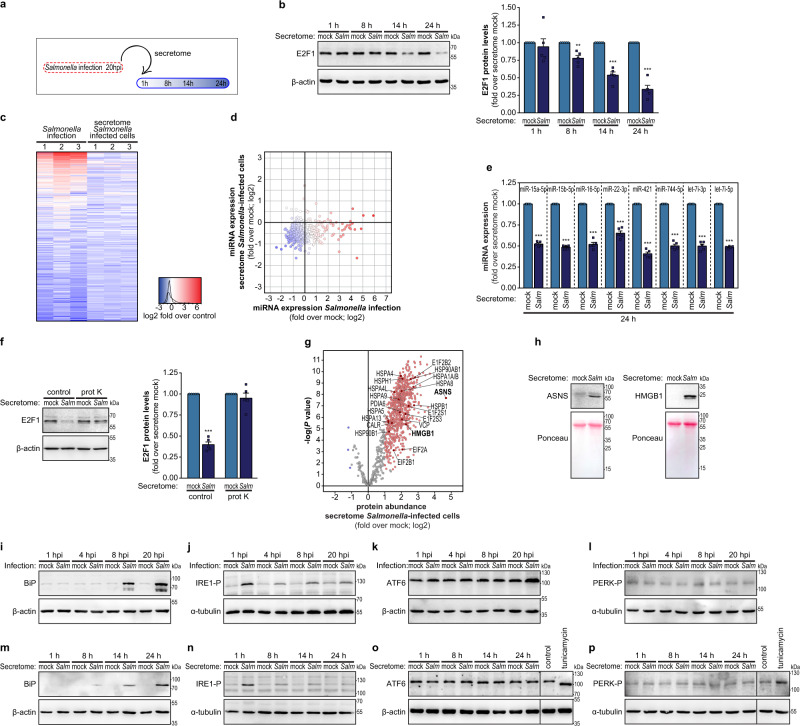
Reduction of E2F1 and miRNA expression is elicited by the secretome of *Salmonella*-infected cells. **a** Schematic representation of the experimental design. Cells were infected with *Salmonella* and the secretome of cells was collected at 20 hpi (unless otherwise indicated). Secretome was centrifuged and filtered through 0.22 µm filters to exclude the presence of bacteria. The secretome was then used to treat naive cells that were analyzed at 1, 8, 14, and 24 h post-treatment. The secretome of mock-treated cells was used for comparison. **b** E2F1 expression, determined by western blot, in naive HeLa cells treated with the secretome of *Salmonella*-infected or mock-treated cells. Results are normalized to naive cells treated with the secretome collected from mock‐treated cells. **c** Heat map showing miRNA expression changes of *Salmonella*-infected cells (same as Fig. 1a) and naive HeLa cells treated with the secretome of *Salmonella*-infected cells (24 h post-treatment). Results are shown as log2 fold change compared to mock-treated cells, or to naive cells treated with secretome collected from mock‐treated cells, respectively. RNA-seq datasets from three independent experiments are shown. Only miRNAs with a number of reads ≥20 in both controls are shown. **d** Comparison of miRNA expression changes in *Salmonella*-infected cells and naive cells treated with the secretome of *Salmonella*-infected cells. Color code for datapoints identical to panel **c**, based on average values. **e** Expression levels of the mature forms of selected miRNAs, determined by qRT-PCR in naive HeLa cells treated with the secretome of *Salmonella*-infected cells, analyzed at 24 h post-treatment. Results are normalized to naive cells treated with secretome collected from mock‐treated cells. **f** E2F1 protein levels, determined by western blot, in naive HeLa cells treated with the secretome of *Salmonella*-infected or mock-treated cells and digested or not with proteinase K, analyzed at 24 h post-treatment. **g** Volcano plot of human proteins identified and quantified in the secretome of *Salmonella*-infected HeLa cells, expressed as log2 fold change compared to the secretome of mock-treated cells, determined by mass spectroscopy analysis. Proteins highlighted in red and blue present significantly increased or decreased in the secretome of infected cells, respectively. Highlighted proteins are associated with ER-stress response. Average from four independent mass spectroscopy experiments are shown. Secretome was collected at 14 hpi. **h** ASNS and HMGB1 protein levels, determined by western blot, in the secretome of *Salmonella*-infected or mock-treated cells, collected at 20 hpi. Ponceau staining of the membranes is shown. **i**–**l** Levels of BiP protein (**i**), IRE1 phosphorylation (**j**), ATF6 protein (**k**), and PERK phosphorylation (**l**), determined by western blot, in HeLa cells infected with *Salmonella* or mock-treated. **m**–**p** Levels BiP protein (**m**), IRE1 phosphorylation (**n**), ATF6 protein (**o**), and PERK phosphorylation (**p**), determined by western blot, in naive HeLa cells treated with the secretome of *Salmonella*-infected or mock-treated cells. Tunicamycin treatment was used as a positive control for ATF6 and PERK activation. *Salmonella* infection was performed at MOI 100. Results are shown as mean ± s.e.m. of *n* = 5 (**b**, **e**, **f**) biologically independent experiments; western blots are representative of *n* = 3 (**h**) or *n* = 5 (**b**, **f**, **i**–**p**) biologically independent experiments; **P < 0.01 and ***P < 0.001 (statistical analysis is detailed in Supplementary Data 1); Source data are provided as a Source Data file.

Considering that extracellular *Salmonella* could not trigger per se E2F1 downregulation in epithelial cells (Supplementary Fig. 1f and ref. ^27^), we reasoned that the reduction of E2F1 expression in bystander cells should likely be prompted by a signaling mechanism between the cells with internalized bacteria and the neighboring bystander cells, presumably mediated by the secretion of extracellular factor(s). In agreement with this hypothesis, treatment of naive HeLa cells with the secretome of *Salmonella*-infected cells (collected at 20 hpi; Fig. 2a) resulted in a strong decrease of E2F1 protein levels, which was particularly noticeable at the later times post-treatment (14 and 24 h; Fig. 2b), when compared to treatment with secretome of mock-treated cells. Similar results were obtained at the mRNA level (Supplementary Fig. 2c). The addition of the supernatant of *Salmonella* cultures (WT, ΔSPI-1, or ΔSPI-2 strains) to naive cells did not affect E2F1 levels (Supplementary Fig. 2d), demonstrating that the secretome of infected cells, and not of the bacteria, is responsible for triggering the observed effects on bystander cells.

Global analysis of miRNA expression revealed that treatment of naive cells with the secretome of *Salmonella*-infected cells results in a reduction of expression of 278 miRNAs (at least 1.4-fold compared with cells treated with secretome of mock-treated cells; 51% of the 542 detected miRNAs) (Fig. 2c). Importantly, 154 (28% of total; 59% of *Salmonella* downregulated) miRNAs were commonly downregulated in naive cells treated with the secretome of *Salmonella*-infected cells, and in cells infected with *Salmonella* (Fig. 2c, d). Similarly, 158 miRNAs were commonly downregulated in bystander cells and in naive cells treated with the secretome of *Salmonella*-infected cells, which corresponds to 64% and 57% of the miRNAs downregulated in either of them, respectively (Supplementary Fig. 2e, f). In agreement with this, treatment of naive cells with the secretome of *Salmonella*-infected cells resulted in decreased expression of the selected panel of E2F1-dependent miRNAs (Fig. 2e). This very strong overlap strongly supports the existence of a common mechanism (E2F1 downregulation) that orchestrates the downregulation of miRNAs in both experimental conditions. Nevertheless, differences between the miRNome profiles of bystander cells and cells treated with secretome can be observed, likely explained by: (i) bystander cells, contrary to cells treated with the secretome, are initially exposed to the bacteria inoculum, even if the bacteria are not internalized or are eliminated from inside the cells in the course of the infection; (ii) the bystander cells are exposed to increasing concentrations of the factors secreted to the extracellular space by infected cells during the 20 h of infection, whereas the naive cells treated with the secretome are exposed to the full secretome for 24 h. As such, the existence of other mechanisms acting on miRNA expression, in addition to E2F1 regulation, cannot be excluded. Regarding the higher number of miRNAs downregulated in E2F1 knockdown cells (245 miRNAs) compared with the miRNAs commonly downregulated in naive cells treated with the secretome and infected cells (154 miRNAs), this is arguably explained by the fact that E2F1 knockdown cells accumulated for a longer time the consequences of decreased levels of the transcription factor. In fact, E2F1 knockdown cells were collected 48 h after siRNA transfection, whereas the *Salmonella*-infected cells were collected at 20 hpi and the cells treated with the secretome were collected 24 h post-treatment. This is particularly pertinent taking into consideration that miRNAs have long half-lives^29,30^.

Overall, these results demonstrate that the secretome of *Salmonella*-infected cells is sufficient to elicit E2F1 and miRNA downregulation of naive cells, explaining a major fraction of the miRNA regulation observed in bystander cells during *Salmonella* infection.

### E2F1 downregulation is triggered by activation of the endoplasmic reticulum stress response, induced by infection or by the secretome of infected cells

To understand whether the decrease of E2F1 expression prompted by the secretome of *Salmonella*-infected cells was due to the secretion of peptide(s)/protein(s), we performed a thorough digestion of the secretome with proteinase K. Interestingly, proteinase K digestion completely impaired the ability of the secretome to trigger E2F1 downregulation (Fig. 2f). In face of this observation, we next analyzed the protein composition of the secretome by mass spectrometry. The analysis was performed for the secretome of infected cells collected at 14 hpi to minimize changes in the secretome triggered by cell death/cytotoxicity associated with the late stages of *Salmonella* infection. The majority of the identified proteins were significantly enriched in the secretome of *Salmonella*-infected cells compared to mock-treated cells (722 proteins; Fig. 2g and Supplementary Data 2). Functional analysis of these proteins revealed enrichment, among others, for proteins associated with the endoplasmic reticulum stress response (ER-stress response, a.k.a. UPR, unfolded protein response; Supplementary Data 3), including asparagine synthetase (ASNS)^31^, high mobility group box1 (HMGB1)^32^, calreticulin^33^, glucose-regulated protein 94 (GRP94, HSP90B1)^34,35^, glucose-regulated protein 75 (GRP75, HSPA9)^35^, binding immunoglobulin protein (BiP, GRP78, HSPA5)^35,36^, and heat-shock protein 110 (HSP110)^37^. The increase of ASNS and HMGB1 levels in the secretome of *Salmonella*-infected cells was validated by western blot (Fig. 2h). Only one *Salmonella* protein was identified in the secretome of infected cells—glycerol-3-phosphate transporter periplasmic binding protein (gene ugpB).

To elucidate the contribution of the ER-stress response to the decrease of E2F1 and miRNA expression during *Salmonella* infection, we first examined whether this pathway is activated upon *Salmonella* infection and/or upon treatment of naive cells with the secretome of *Salmonella*-infected cells. We observed that ER-stress response is indeed activated in both conditions, as assessed by the increased expression of the ER chaperone BiP^36^ (Fig. 2i, m) and phosphorylation of the ER-stress sensor IRE1 (ref. ^38^) (Fig. 2j, n and Supplementary Fig. 2g, h). IRE1 phosphorylation occurs very early on during infection (1 hpi) or secretome treatment (1 h), and although phosphorylation decreases with time, it is maintained at high levels compared to control cells (Fig. 2j, n and Supplementary Fig. 2g, h). The increase of BiP occurred later than IRE1 phosphorylation (starting at 8 hpi or 8 h post-treatment with the secretome). This is consistent with the increase of BiP protein levels occurring only after prolonged ER stress^39^. Interestingly, ER-stress response was only triggered by infection or treatment with the secretome of invasive *Salmonella* strains (WT and ΔSPI-2), but not with the non-invasive strain ΔSPI-1 (Supplementary Fig. 2i, j), suggesting a causal relationship between ER-stress response and E2F1/miRNA regulation. Of note, *Salmonella* infection or treatment with the secretome of *Salmonella*-infected cells did not activate the two additional ER-stress sensors—ATF6 (Figs. 2k, o) and PERK (Fig. 2l, p).

**Fig. 3:**
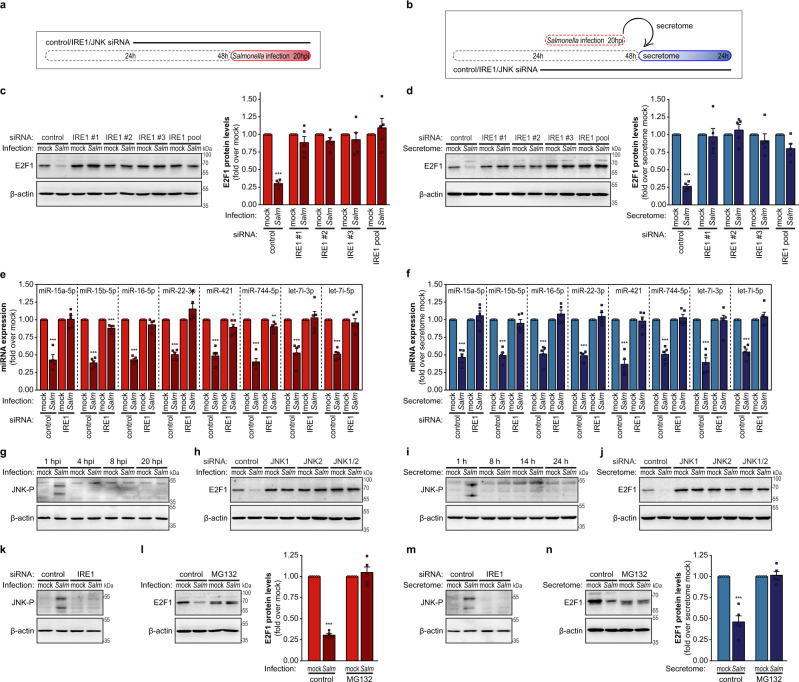
Activation of the ER-stress response pathway by *Salmonella* infection or by the secretome of infected cells leads to E2F1 downregulation. **a** Schematic representation of the experimental design for infection experiments. Cells were transfected with control, IRE1, or JNK siRNAs, and 48 h later were infected with *Salmonella*. Samples were analyzed at 1 and 20 hpi. **b** Schematic representation of the experimental design for secretome treatment. Cells were transfected with control, IRE1, or JNK siRNAs, and 48 h later were treated with the secretome of mock-treated or *Salmonella*-infected cells collected at 20 hpi. Samples were analyzed at 1 and 24 h post-treatment. **c**, **d** E2F1 protein levels, determined by western blot, in HeLa cells transfected with control, three independent siRNAs or a pool of siRNAs targeting IRE1, and either infected with *Salmonella* or mock-treated (20 hpi; **c**) or treated with the secretome of *Salmonella*-infected cells or mock-treated cells (24 h; **d**). **e**, **f** Expression levels of selected miRNAs, determined by qRT-PCR, in HeLa cells transfected with control or IRE1 siRNAs, and infected with *Salmonella* or mock-treated (20 hpi; **e**) or treated with the secretome of *Salmonella*-infected cells or mock-treated cells (24 h; **f**). Results are normalized to mock‐treated cells. **g**, **i** JNK phosphorylation, determined by western blot, in HeLa cells either infected with *Salmonella* or mock-treated (**g**) or treated with the secretome of *Salmonella*-infected cells or mock-treated cells (**i**). **h**, **j** E2F1 protein levels, determined by western blot, in HeLa cells transfected with control, JNK1, JNK2, or JNK1/JNK2 siRNAs, and either infected with *Salmonella* or mock-treated (20 hpi; **h**) or treated with the secretome of *Salmonella*-infected cells or mock-treated cells (24 h; **j**). **k**, **m** JNK phosphorylation, determined by western blot, in HeLa cells transfected with control or IRE1 siRNAs, and either infected with *Salmonella* or mock-treated (1 hpi; **k**) or treated with the secretome of *Salmonella*-infected cells or mock-treated cells (1 h; **m**). **l**, **n** E2F1 protein levels, determined by western blot, in HeLa cells treated with the proteasome inhibitor MG132 or control (DMSO), and either infected with *Salmonella* or mock-treated (20 hpi; **l**) or treated with the secretome of *Salmonella*-infected cells or mock-treated cells (24 h; **n**). *Salmonella* infection was performed at MOI 100. Results are shown as mean ± s.e.m. of *n* = 5 (**c**, **e**, **f**, **l**, **n**) or *n* = 6 (**d**) biologically independent experiments; western blots are representative of *n* = 3 (**g**, **i**, **k**, **m**), *n* = 5 (**c**, **h**, **j**, **l**, **n**) or *n* = 6 (**d**) biologically independent experiments; *P < 0.05, **P < 0.01, and ***P < 0.001 (statistical analysis is detailed in Supplementary Data 1); Source data are provided as a Source Data file.

The effect of ER-stress response as a trigger for E2F1 regulation was confirmed by the strong decrease of E2F1 levels observed in HeLa cells treated with well-described inducers of ER stress, specifically DTT and tunicamycin (Supplementary Fig. 3a), in agreement with a previous report^40^. Importantly, knockdown of IRE1 (siRNA pool and three independent siRNAs) or inhibition of its kinase activity by the chemical inhibitor KIRA6 (ref. ^41^) strongly impaired the E2F1 downregulation prompted by *Salmonella* infection (Fig. 3a, c and Supplementary Fig. 3b, d) or by treatment of naive cells with the secretome of *Salmonella*-infected cells (Fig. 3b, d and Supplementary Fig. 3c, e). Moreover, preventing the activation of the IRE1 branch of the UPR pathway also compromised the downregulation of the selected miRNAs (miR-15a-5p, miR-15b-5p, miR-16-5p, miR-22-3p, miR-421, miR-744-5p, let-7i-3p, and let-7i-5p) (Fig. 3e, f and Supplementary Fig. 3f, g). Interestingly, ASNS levels were dramatically reduced in the secretome of IRE1 knockdown cells infected with *Salmonella*, when compared to the secretome of control-infected cells (Supplementary Fig. 3h). This result demonstrates that in our experimental setup ASNS induction is dependent on the IRE1 branch of the UPR. Of note, knockdown of ATF6 or PERK did not affect E2F1 downregulation elicited by *Salmonella* infection or secretome treatment (Supplementary Fig. 3i, j), indicating that neither of these branches contributes to E2F1 regulation in these biological settings. The efficiency of IRE1, ATF6, and PERK knockdown and inhibition of IRE1 kinase activity by KIRA6 were confirmed by western blot (Supplementary Fig. 3k–n). IRE1 knockdown efficiency was further demonstrated by the impaired splicing of XBP1 upon tunicamycin treatment (Supplementary Fig. 3o), which is dependent on IRE1 endonuclease activity^42^. Finally, we confirmed that activation of the ER-stress response pathway induced by treatment with the secretome of *Salmonella*-infected cells was impaired in IRE1 knockdown cells or KIRA6-treated cells (Supplementary Fig. 3p, q**)**, as evaluated by BiP expression.

The *Salmonella* effector protein SlrP has been shown to partially co-localize and interact with the ER chaperone ERdj3, reducing the interaction of ERdj3 with denatured proteins, suggesting that it could increase the accumulation of unfolded proteins in the ER, and thus activate ER-stress response^43^. However, no differences in IRE1 activation and E2F1 downregulation were observed between infection with WT or Δ*slrP Salmonella* strains (Supplementary Fig. 4a, b), excluding this effector protein as the trigger for ER-stress activation.

Collectively, these results demonstrate that the IRE1 branch of the ER-stress response pathway is triggered during *Salmonella* infection of epithelial cells and that the secretome of *Salmonella*-infected cells is sufficient to induce this phenotype in naive cells. Importantly, the activation of the IRE1 pathway triggers E2F1 and miRNA downregulation.

### IRE1 activation induced by infection or by the secretome of infected cells leads to JNK pathway activation and subsequent E2F1 proteasomal degradation

Following activation, the IRE1 endoribonuclease activity processes the mRNA encoding the transcription factor XBP1, leading to the expression of an active transcription factor (XBP1s) that transactivates a subset of UPR target genes^44^. IRE1 also degrades specific mRNAs through a process denominated regulated IRE1-dependent decay (RIDD)^45–47^. In addition, it is well described that the TNF receptor-associated factor 2 (TRAF2) protein binds to phosphorylated IRE1, facilitating the recruitment and activation of the JNK MAPK pathway^48^.

Monitoring of downstream targets of IRE1 activation, specifically splicing of XBP1 and expression levels of typical IRE1/XBP1 targets (e.g. ERdj4/DNAJB9, EDEM1, SEC24D) by qRT-PCR in *Salmonella*-infected cells and naive cells treated with secretome of *Salmonella*-infected cells revealed differential splicing of XBP1. Interestingly, XBP1 splicing and the consequent activation of downstream targets did not occur in *Salmonella-*infected cells (Supplementary Fig. 4c, d); conversely, both XBP1 splicing and activation of its target genes occurred in naive cells treated with secretome of infected cells (Supplementary  4f, g). The result in *Salmonella*-infected cells is in apparent contradiction with our observation that IRE1 activation occurs in both experimental conditions (demonstrated by IRE1 phosphorylation; Fig. 2j, n). However, we determined that the expression of the RNA ligase RTCB, responsible for the XBP1 exon ligation^49–51^, is markedly decreased in *Salmonella*-infected cells, while it is maintained in cells treated with the secretome (Supplementary Fig. 4e, h). These data suggest that, although IRE1 activation occurs in both conditions, XBP1 splicing is impaired in *Salmonella*-infected cells presumably due to altered expression of the cytoplasmic splicing machinery.

We have clearly demonstrated that IRE1 activation is crucial for E2F1 decreased expression during *Salmonella* infection and secretome treatment (both in IRE1 knockdown cells and cells treated with the IRE1 kinase inhibitor KIRA6; Fig. 3c, d and Supplementary Fig. 3d, e). However, the lack of XBP1 splicing in *Salmonella*-infected cells (Supplementary Fig. 4c, d**)**, as well as the observation that the inhibition of IRE1 endonuclease activity, using the 4µ8C inhibitor^52^, did not affect the extent of E2F1 downregulation observed upon *Salmonella* infection or by treatment with the secretome of *Salmonella*-infected cells (Supplementary Fig. 4i, j) led us to hypothesize that IRE1 could act through a mechanism independent of its endonuclease activity. Of note, the efficiency of the 4µ8C inhibitor was confirmed by quantifying XBP1 splicing in cells treated with tunicamycin (Supplementary Fig. 4k).

According to the literature, the activation of the JNK pathway by the IRE1 pathway is independent of its endonuclease activity^48^. It is already described that the JNK pathway is activated during *Salmonella* infection of epithelial cells^53,54^, independently of innate immune receptors such as TLRs. Our data confirm that this occurs in a time frame compatible with IRE1 activation (Fig. 3g). Moreover, JNK activation also occurs in cells treated with the secretome of *Salmonella*-infected cells (Fig. 3i). Adding to the importance of JNK, we also demonstrate that JNK knockdown prevents the E2F1 downregulation observed during *Salmonella* infection or secretome treatment (Fig. 3h, j and Supplementary Fig. 4l–n). Moreover, in both conditions JNK activation is not detected in IRE1 knockdown cells (Fig. 3k, m). Together, these results demonstrate that JNK activation mediates the E2F1 regulation during *Salmonella* infection/secretome treatment.

Regarding the mechanism of degradation of E2F1 upon *Salmonella* infection or secretome treatment, we tested the relevance of proteasome degradation to this process. Inhibition of the proteasome by MG132 treatment prevented the E2F1 downregulation elicited by *Salmonella* infection or secretome treatment (Fig. 3l, n). Of note, it is described that E2F1 regulates its own promoter activity^55^ and therefore the decrease in the E2F1 protein level likely explains the decrease observed at the mRNA level (Supplementary Figs. 1b, d and 2c).

Overall, our data indicate that IRE1 activation upon *Salmonella* infection or secretome treatment leads to JNK activation, which phosphorylates^56,57^ and thus inactivates E2F1. The inactive transcription factor is then degraded by the proteasomal machinery.

### Secretome of *Salmonella*-infected cells leads to RAGE-dependent ER-stress response pathway activation

**Fig. 4:**
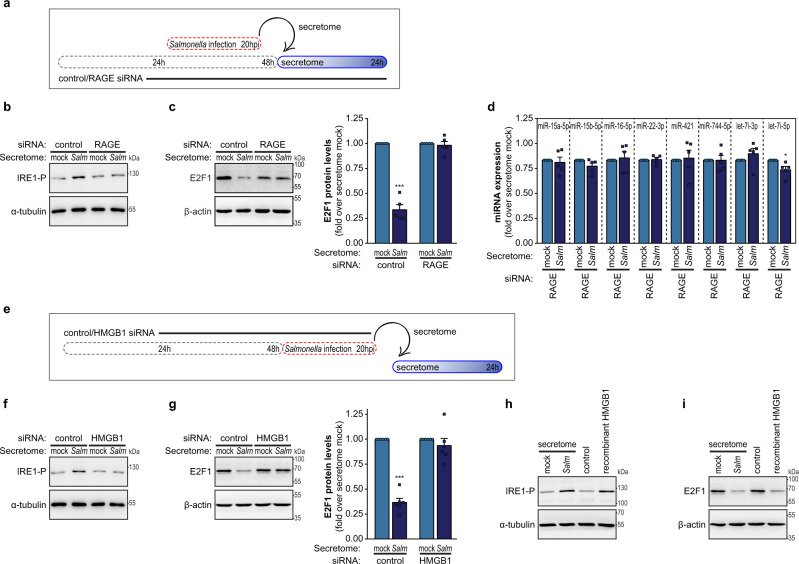
Regulation of E2F1 by the secretome of *Salmonella*-infected cells is triggered by engagement of HMGB1 with the receptor RAGE. **a** Schematic representation of the experimental design for secretome treatment. Cells were transfected with control or RAGE siRNAs and 48 h later were treated with the secretome of mock-treated or *Salmonella*-infected cells collected at 20 hpi. **b**, **c** IRE1 phosphorylation (**b**) and E2F1 protein levels (**c**), determined by western blot, in HeLa cells transfected with control or RAGE siRNAs, and treated with the secretome of *Salmonella*-infected cells or mock-treated cells for 1 or 24 h, respectively. **d** Expression levels of selected miRNAs, determined by qRT-PCR, in HeLa cells transfected with RAGE siRNA, and treated with the secretome of *Salmonella*-infected cells or mock-treated cells for 24 h. Results are normalized to naive cells treated with secretome collected from mock‐treated cells. Compare with HeLa cells transfected with control siRNA (Fig. 3f). **e** Schematic representation of the experimental design for the treatment of naive cells with the secretome of HMGB1-depleted cells. Secretome of cells transfected with control or HMGB1 siRNAs and *Salmonella*-infected or mock-treated were used to treat naive cells. **f**, **g** IRE1 phosphorylation (**f**) and E2F1 protein levels (**g**), determined by western blot, in naive HeLa cells treated with the secretome of *Salmonella*-infected or mock-treated HMGB1-depleted cells for 1 or 24 h, respectively. **h**, **i** IRE1 phosphorylation (**h**) and E2F1 protein levels (**i**), determined by western blot, in HeLa cells treated with recombinant HMGB1 protein for 1 or 24 h, respectively. Naive cells treated with the secretome of *Salmonella*-infected cells or mock-treated cells are shown for comparison. *Salmonella* infection was performed at MOI 100. Results are shown as mean ± s.e.m. of *n* = 5 (**c**, **d**, **g**) biologically independent experiments; western blots are representative of *n* = 3 (**b**, **f**, **h**, **i**) or *n* = 5 (**c**, **g**) biologically independent experiments; *P < 0.05 and ***P < 0.001 (statistical analysis is detailed in Supplementary Data 1); Source data are provided as a Source Data file.

Considering that the protein HMGB1 is highly enriched in the secretome of *Salmonella*-infected cells (Fig. 2g, h) and that this protein has been shown to activate the ER-stress response by a mechanism dependent on its interaction with RAGE^58–60^, we evaluated the involvement of RAGE in the downregulation of E2F1 (Fig. 4a**)**. Firstly, we confirmed that activation of the ER-stress response pathway elicited by treatment with the secretome of *Salmonella*-infected cells was impaired in RAGE knockdown cells, as evaluated by IRE1 phosphorylation status and/or BiP expression (Fig. 4b and Supplementary Fig. 5a). Importantly, the knockdown of RAGE also prevented the E2F1 downregulation upon treatment with the secretome of infected cells (Fig. 4c). Concurring with these results, the downregulation of the panel of selected miRNAs upon treatment with the secretome of *Salmonella*-infected cells was impaired in RAGE knockdown cells (Fig. 4d). Moreover, IRE1 activation and E2F1 downregulation did not occur in naive cells treated with the secretome of *Salmonella*-infected HMGB1-depleted cells (Fig. 4e–g). Accordingly, treatment of naive cells with recombinant HMGB1 induced both IRE1 activation and consequent decrease of E2F1 levels (Fig. 4h, i). Efficient knockdown of RAGE or HMGB1 was confirmed by western blot (Supplementary Fig. 5b, c).

Overall, these results demonstrate that the ER-stress response activation and consequent E2F1 regulation induced by the secretome of *Salmonella*-infected cells is dependent on the engagement of RAGE, elicited by the HMGB1.

### Downregulation of E2F1 promotes *Salmonella* infection

**Fig. 5:**
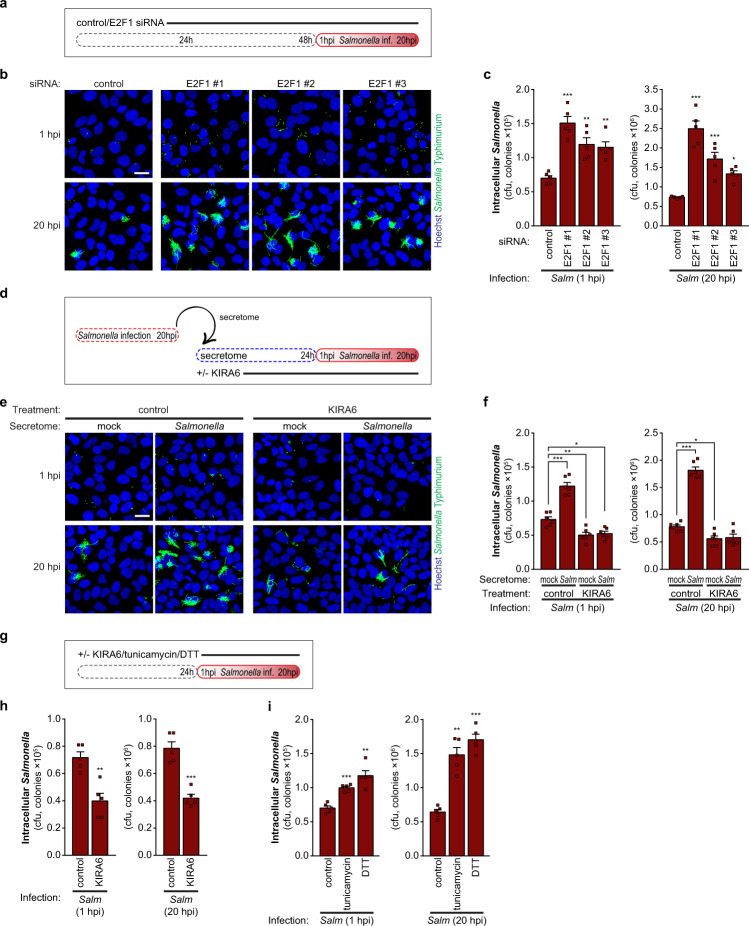
E2F1 downregulation promotes *Salmonella* infection. **a** Schematic representation of the experimental design. Cells were transfected with control or three independent siRNAs targeting E2F1 and 48 h later cells were infected with *Salmonella*. Infection was analyzed at 1 and 20 hpi. **b**, **c** Representative images (**b**) and quantification of intracellular *Salmonella* by cfu (**c**) of HeLa cells transfected with control or E2F1 siRNAs, and infected with *Salmonella*. Scale bar, 25 µm. **d** Schematic representation of the experimental design. The secretome of *Salmonella*-infected cells was used to treat naive cells for 24 h with control (DMSO) or KIRA6. Cells were then infected with *Salmonella* and analyzed at 1 and 20 hpi. KIRA6/control treatment was maintained during infection. The secretome of mock-treated cells was used for comparison. **e**, **f** Representative images (**e**) and quantification of intracellular *Salmonella* by cfu (**f**) of naive HeLa treated with the secretome of *Salmonella*-infected cells or mock-treated cells, treated with KIRA6/control, and then infected with *Salmonella*. Scale bar, 25 µm. **g** Schematic representation of the experimental design. Cells were pre-treated for 24 h with control (DMSO), the chemical inhibitor of IRE1 kinase activity KIRA6, or the ER-stress inducers tunicamycin or DTT and then infected with *Salmonella*. Treatments were maintained during infection. Infection was analyzed at 1 and 20 hpi. **h**, **i** Quantification of intracellular bacteria by cfu of HeLa cells infected with *Salmonella*, upon treatment with control/KIRA6 (**h**) or control/tunicamycin/DTT (**i**). Infection was performed at MOI 10. Results are shown as mean ± s.e.m. of *n* = 5 (**c**, **h**, **i**) or *n* = 6 (**f**) biologically independent experiments; microscopy images are representative of *n* = 3 (**b**, **e**) biologically independent experiments; *P < 0.05, **P < 0.01, and ***P < 0.001 (statistical analysis is detailed in Supplementary Data 1).

Considering the magnitude of E2F1 downregulation and its specificity during *Salmonella* infection, we pondered whether it could impact the infection process. Indeed, E2F1 knockdown increased *Salmonella* infection, specifically the bacterial invasion (1 hpi) and replication (20 hpi), compared to cells transfected with control siRNA (Fig. 5a–c). Moreover, cells pre-treated with secretome from *Salmonella*-infected cells also presented increased bacterial invasion and replication when compared to cells pre-treated with secretome from mock-treated cells (Fig. 5d–f).

Conversely, preventing E2F1 downregulation by blocking the activation of the IRE1 pathway (KIRA6 treatment) inhibited *Salmonella* infection (Fig. 5g, h) and thwarted the increase in *Salmonella* infection elicited by pre-treatment with the secretome of *Salmonella*-infected cells (Fig. 5d–f). Moreover, when the ER-stress response was prompted independently of infection, by tunicamycin or DTT treatment, an increase of *Salmonella* invasion and replication was observed when compared to control cells (Fig. 5g, i).

Together, these results show that the decrease of E2F1 expression upon *Salmonella* infection that ensues from activation of the ER-stress response pathway is required to sustain a productive bacterial replication inside host cells, as well as to promote infection of bystander cells.

### *Salmonella* infection triggers IRE1 activation and E2F1 downregulation in vivo

**Fig. 6:**
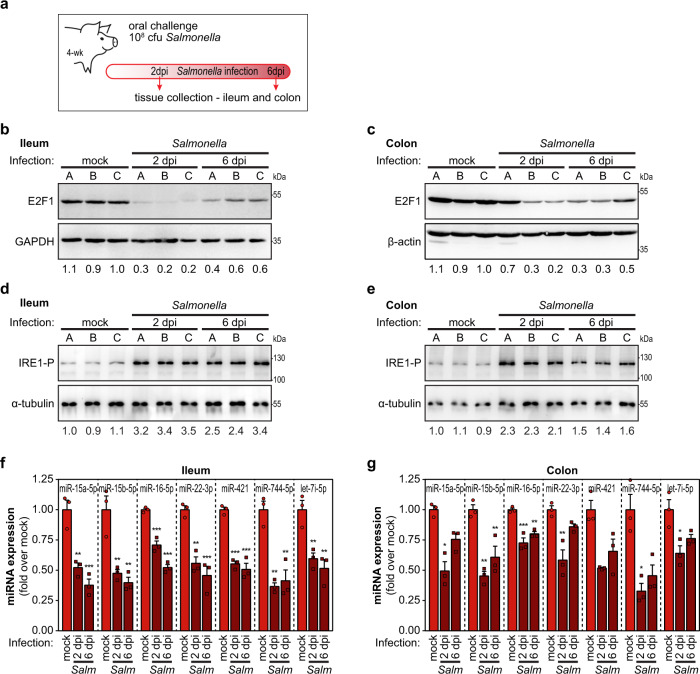
E2F1 and miRNA downregulation upon *Salmonella* infection also occurs in vivo. **a** Schematic representation of the in vivo *Salmonella* infection experiments. Four-week-old piglets were orally challenged with *Salmonella*; tissue samples of ileum and colon were collected at 2 and 6 days post-infection (dpi). Samples of untreated animals (mock) were collected for comparison. **b**–**e** Levels of E2F1 protein (**b**, **c**) and IRE1 phosphorylation (**d**, **e**), evaluated by western blot, in ileum and colon samples obtained from untreated animals (mock) or piglets challenged with *Salmonella*. Values below the western blots indicate E2F1:GAPDH, E2F1:β-actin, or IRE1-P:α-tubulin ratios; averages of the three control samples were set to 1. **f**, **g** Expression levels of the mature forms of selected miRNAs, determined by qRT-PCR, in the ileum (**f**) and colon (**g**) samples obtained from untreated animals (mock) or piglets challenged with *Salmonella*. Three animals were analyzed per condition/time point. Results are shown as mean ± s.e.m.; *P < 0.05, **P < 0.01, and ***P < 0.001 (statistical analysis is detailed in Supplementary Data 1); Source data are provided as a Source Data file.

Having shown that *Salmonella* infection elicits a strong downregulation of E2F1 in vitro with important consequences for the outcome and dissemination of infection, we examined whether E2F1 regulation could be observed in vivo. We used a piglet model of *Salmonella* infection and compared E2F1 expression in mucosal samples of ileum and colon tissues of control or *Salmonella*-infected animals, collected at 2 and 6 days post-infection (Fig. 6a). The levels of E2F1 were strongly decreased in the ileum and colon samples from *Salmonella*-infected animals compared to control animals (Fig. 6b, c). Conversely, and in agreement with the data obtained in in vitro infection models, IRE1 phosphorylation was significantly increased in tissues collected from infected animals (Fig. 6d, e).

Moreover, analysis of expression of the subset of selected miRNAs, specifically miR-15a-5p, miR-15b-5p, miR-16-5p, miR-22-3p, miR-421, miR-744-5p, and let-7i-5p, revealed that these miRNAs are downregulated in infected piglets, when compared to control animals, both in ileum and colon samples (Fig. 6f, g). Along this line, we have previously shown that let-7i-3p expression is decreased in ileum and colon of infected piglets and that the expression of let-7i-3p direct target RGS2 is increased^28^.

Overall, these results demonstrate that also in an animal model, *Salmonella* infection results in a strong downregulation of E2F1 expression, likely triggered by activation of the IRE1 branch of the ER-stress response pathway, as well as the consequent downregulation of E2F1-dependent miRNAs further supporting their relevance for the infection process.

## Discussion

**Fig. 7:**
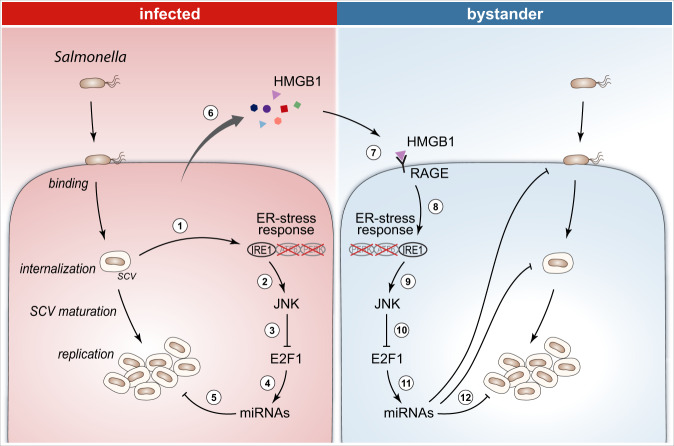
Model depicting the role of E2F1 in *Salmonella* infection and dissemination to bystander cells. *Salmonella* internalization activates the ER-stress response (1), specifically the IRE1 branch, which activates the JNK MAPK pathway (2) leading to E2F1 downregulation (3) and a consequent decrease in expression of a significant number of miRNAs (4). Given that many of these miRNAs were shown to counteract infection, the net outcome of miRNA regulation is an increase of *Salmonella* intracellular replication (5). Infection of cells by *Salmonella* leads to increased secretion of proteins to the extracellular milieu (several of which are related to the ER-stress response), including HMGB1 (6). HMGB1 binds to the RAGE receptor at the surface of bystander cells (7), triggering an IRE1-mediated ER-stress response in bystander cells (8). This, in turn, activates the JNK pathway (9) resulting in a decrease of E2F1 expression in bystander cells (10). Echoing the effect in infected cells, E2F1 downregulation decreases the expression of numerous miRNAs (11), with a net effect of priming these cells for *Salmonella* infection, by favoring bacterial binding to bystander cells, as well as by promoting bacterial internalization and intracellular replication (12).

Although miRNAs are increasingly recognized for their crucial role in the interaction between host and bacterial pathogens, the exact mechanisms underlying their regulation during infection remain poorly understood. In this study, we identified and characterized the transcription factor E2F1 as a major player in the regulation of miRNAs upon *Salmonella* infection of epithelial cells (Fig. 7). On a global scale, E2F1-dependent miRNAs account for more than 50% of the miRNAs downregulated during *Salmonella* infection. *Salmonella* infection elicits a strong decrease of E2F1 expression, which appears to be restricted to this pathogen, not occurring in response to infection by other intracellular pathogens (*Shigella flexneri* and *Listeria monocytogenes*). Of note, E2F1 downregulation is dependent on bacterial invasion rather than on extracellular *Salmonella*, given that purified LPS, heat-killed *Salmonella*, and the invasion-deficient ΔSPI-1 *Salmonella* mutant failed to induce regulation. These results clearly establish E2F1 regulation upon *Salmonella* infection as a specific effect, rather than a broad and/or unspecific response to bacterial components/infection.

Interestingly, we demonstrate that E2F1 downregulation and subsequent miRNA regulation is not limited to infected cells, but it also occurs, and to a remarkably comparable extent, in bystander cells. This is particularly interesting in light of previous studies focused on various intracellular bacterial pathogens that revealed that, far from being passive observers, bystander cells play a critical role in controlling infection^7–9^. Noteworthy, our study proposes a shift of the current paradigm by demonstrating that changes elicited in bystander cells can positively contribute to the progression of infection. Indeed, we demonstrate that E2F1 downregulation in bystander cells renders these cells more permissive to infection, by promoting *Salmonella* invasion, and intracellular replication. The net effect of E2F1 and miRNA downregulation resulting in increased *Salmonella* infection is likely explained by the fact that several of these miRNAs have been shown to counteract *Salmonella* infection. Based on a genome-wide microscopy-based functional screening approach we have identified miRNAs controlling infection of epithelial cells by *Salmonella*^27,28^. Among others, the miRNAs selected for validation in the present study, namely miR-15a-5p, miR-15b-5p, miR-16-5p, miR-22-3p, miR-421, miR-744-5p, let7i-3p, and let-7i-5p, are among the miRNAs strongly inhibiting *Salmonella* infection. Of note, miRNA independent effects of E2F1 downregulation may also contribute to the observed increase of *Salmonella* infection.

An aspect of particular relevance is the trigger/signal that mediates the signaling between infected and bystander cells, ultimately leading to E2F1 and miRNA downregulation. The mechanism of intercellular communication is determinant to the range of action of the signal: signals transmitted only to cells in the immediate vicinity of the infected cell likely rely on direct cell-to-cell communication via gap junctions, whereas long-range communication likely reflects the release of soluble signals or secretion of exosomes or microvesicles. Taking into consideration that the extent of E2F1 downregulation was comparable in infected and bystander cells, and the relatively low percentage of *Salmonella-*infected cells (25–30%) in the experimental conditions tested, we reasoned that a long-range communication mechanism was likely involved. This was unequivocally demonstrated by showing that the secretome of *Salmonella*-infected cells, in particular proteins present therein, is sufficient to trigger E2F1 downregulation and consequent miRNA regulation in naive cells.

Analysis of the protein composition of the secretome by mass spectrometry revealed a dramatic enrichment of protein abundance in the secretome of *Salmonella*-infected cells, when compared to that of mock-treated cells. Importantly, there were several proteins previously shown to be involved in or secreted upon activation of the ER-stress response, including asparagine synthetase (ASNS)^31^, the most highly enriched protein in the secretome of infected cells. Based on this, we hypothesize that ER-stress response could be induced in *Salmonella*-infected cells. It has been well described that ER-stress response promotes inflammatory pathways and is involved in a wide range of related pathologies, such as diabetes, obesity, inflammatory bowel disease, and atherosclerosis^61,62^. Recent evidence has also implicated this pathway in the infection by bacterial pathogens^63–66^. Although the activation or inhibition of the ER-stress response has been reported for several bacterial pathogens (e.g. *Brucella abortus*^67^, *Chlamydia pneumoniae*^68^, group A *Streptococcus*^69^, *Campylobacter jejuni*^70^, *Legionella pneumophila*^71^) in most cases its role for pathogenesis remains unclear. Here, we demonstrate that the activation of the ER-stress response, and consequent E2F1 and miRNA downregulation, is beneficial for *Salmonella* replication and dissemination of infection. Particularly, we showed that ER-stress response is activated both in *Salmonella*-infected cells and in cells treated with the secretome of infected cells. Most importantly, we demonstrated that E2F1 downregulation is triggered by ER-stress response, establishing a causal relationship between induction of ER-stress response in infected/bystander cells and increased *Salmonella* infection.

Mechanistically, we show that *Salmonella* infection and treatment with secretome of infected cells specifically activates the ER-stress sensor IRE1, whereas the two other ER-stress response branches mediated by ATF6 and PERK are not activated (Fig. 7). Our results are in apparent contradiction with a previous report indicating that ER-stress response, specifically the PERK branch, is triggered during *Salmonella* infection of mice^72^. This can potentially be explained by the different organisms evaluated, namely mouse tissue^72^ versus human cells and piglet tissue (this study), and/or infection procedure/protocol. As such, it is possible that in distinct cellular/organism models other branches of ER-stress response pathway are activated upon *Salmonella* infection. Following IRE1 activation upon *Salmonella* infection/secretome treatment, our data support a model in which the JNK pathway is activated by IRE1 leading to phosphorylation of E2F1, among other targets. The inactive E2F1 is then degraded by proteasomal degradation (Fig. 7). JNK was previously shown to phosphorylate E2F1, inactivating the transcription factor^56,57^.

Regarding the stimulation of ER-stress response in naive cells upon treatment with the secretome of *Salmonella*-infected cells, we identified the activation of RAGE transmembrane receptor as the trigger for the induction of ER-stress response. RAGE activation has been previously shown to mediate ER-stress response in various cell types^58–60,73–75^, upon interaction with various ligands including the protein HMGB1 (refs. ^58–60^). In the case of *Salmonella* infection, our results demonstrate that RAGE activation is engaged by the binding of the protein HMGB1. Indeed, we show that HMGB1 is secreted to the extracellular milieu by *Salmonella*-infected epithelial cells, and that this secreted protein is both required and sufficient to bystander cell activation. Of note, it has been previously shown that HMGB1 is released to the intestine of piglets infected with *Salmonella*^76^, corroborating our in vitro observations and our in vivo data showing activation of ER-stress response in intestinal samples of *Salmonella*-infected piglets.

IRE1 has been shown to cleave precursor miRNAs, specifically those of miR-17, miR-34a, miR-96, and miR-125a, mediating their degradation^77^. As such, it is conceivable that IRE1-mediated degradation directly regulates the levels of these specific miRNAs during infection. However, it is not likely that IRE1-mediated degradation is contributing to the regulation of the high number of miRNAs downregulated during *Salmonella* infection, given that the effect of IRE1 on miRNAs appears to be specific to motifs present in those particular precursor miRNAs^77^. It is conceivable that for miR-34a and miR-125a-5p, miRNAs that are not significantly changed in E2F1 knockdown cells but that are downregulated during *Salmonella* infection and secretome treatment, there is a contribution of IRE1-mediated degradation.

In summary, we demonstrate that downregulation of the transcription factor E2F1 and consequent miRNome changes are crucial for *Salmonella* infection, by promoting *Salmonella* replication in infected cells and priming bystander cells for more efficient bacterial infection. Moreover, our study challenges the current paradigm by demonstrating that reprogramming of bystander cells during bacterial infection can positively contribute to the dissemination of infection.

## Methods

### Mammalian cell culture

Human epithelial HeLa-229 (ATCC CCL-2.1) were cultured in DMEM GlutaMAX containing 1.0 g/l glucose (Gibco, 21885), and human colon cancer HCT-8 (ATCC CCL-244) were cultured in RPMI 1640 GlutaMAX (Gibco, 72400). Cell lines were acquired from ATCC/LGC Standards and no further authentication was performed. Media were supplemented with 10% fetal bovine serum (Biochrom, S0115). Cells were maintained at 37 °C in a 5% CO_2_ humidified atmosphere. All cell lines tested negative for mycoplasma contamination.

For confocal microscopy, cfu assays, and RNA isolation experiments, cells were seeded 48 h before infection in 24-well plates, at a density of 6.0 × 10^4^ (HeLa) or 8.0 × 10^4^ (HCT-8) cells per well; for western blot and secretome collection, cells were seeded 48 h before infection in six-well plates, at a density of 2.4 × 10^5^ (HeLa) or 3.2 × 10^5^ (HCT-8) cells per well.

For LPS stimulation, cells were treated with LPS purified from *Salmonella* Typhimurium (Sigma, L6143) at 1 and 10 μg/ml for 24 h.

For treatment with ER-stress-inducing compounds, cells were incubated with DTT (1 mM; Roth, 6908.4) or tunicamycin (0.5 μg/ml; Sigma, T-7765) for 24 h. For infection, cells were treated with DTT and tunicamycin for 24 h prior to infection and during infection (with 10 μg/ml gentamicin). For XBP1 splicing analysis, cells were treated with tunicamycin (2 μg/ml) for 6 h.

For IRE1 kinase activity inhibition, cells were treated with the KIRA6 (0.5 µM; Merck, 5322810001) as follows: for 24 h prior to infection and during the infection (with 10 μg/ml gentamicin); for 24 h during secretome treatment and during the infection (with 10 μg/ml gentamicin); for 24 h during the secretome treatment. For IRE1 endonuclease activity inhibition, cells were treated with 4μ8C (Merck, 412512) for 1 h prior to infection/secretome treatment (50 μM) and during infection (with 10 μg/ml gentamicin) or secretome treatment for 24 h (25 μM). For XBP1 splicing analysis, cells were treated with 4μ8C for 1 h (50 μM) and then simultaneously treated with 4μ8C (25 μM) and tunicamycin (2 μg/ml) for 6 h.

For recombinant HMGB1 treatment, cells were incubated with 10 μg/ml of hHMGB1 (Sigma, SRP6265) for 1 or 24 h.

For inhibition of the proteasome, cells were treated with MG132 (20 μM; Sigma, 474787) during infection (with 10 μg/ml gentamicin) and for 24 h during the secretome treatment.

### Bacterial strains

*Salmonella enterica* serovar Typhimurium strain SL1344 expressing GFP constitutively from a chromosomal locus^78^, *Shigella flexneri* serotype 5 strain M90T, and *Listeria monocytogenes* serovar 1/2a EGD-e were used in this study. The *Salmonella* ΔSPI-1, ΔSPI-2, and Δ*slrP* mutant strains were previously described^79^. For piglet infections, *Salmonella enterica* serovar Typhimurium phagetype DT104 isolated from a carrier pig^80^ was used. *Shigella* and *Salmonella* were grown aerobically in Luria broth (LB) medium, and *Listeria* was grown in Brain Heart Infusion (BHI) medium. When appropriate, the medium was supplemented with the following antibiotics: ampicillin 100 μg/ml, chloramphenicol 20 μg/ml, kanamycin 25 μg/ml.

### Bacterial infections

For *Salmonella*, *Shigella*, and *Listeria* infections, overnight cultures were diluted 1:100 in LB (*Salmonella* or *Shigella*) or 1:50 in BHI medium (*Listeria*) and grown at 37 °C with shaking until OD_600_ 2 (*Salmonella*), OD_600_ 0.4 (*Shigella*), or OD_600_ 0.7 (*Listeria*). Bacteria were then harvested by centrifugation for 2 min at 12,000*g* and resuspended in complete medium. Cells were infected with the bacteria at the MOI indicated in the figure/figure legend. After the addition of bacteria, cells were centrifuged at room temperature (RT) for 10 min at 250*g* (*Salmonella* or *Listeria*) or for 15 min at 2000*g* (*Shigella*) and incubated at 37 °C in a 5% CO_2_ humidified atmosphere for 20 min (*Salmonella* or *Listeria*) or 15 min (*Shigella*). Extracellular bacteria were killed by replacing the medium with fresh medium containing 50 μg/ml gentamicin for 30 min. The medium was then exchanged to medium containing 10 μg/ml gentamicin, until analysis.

The fraction of *Salmonella*-infected cells was determined by flow cytometry and by fluorescence microscopy followed by automated image analysis. In the experimental conditions used for the majority of the experiments in the manuscript, specifically infection of HeLa cells with *Salmonella* at MOI 100, both analyses show that the percentage of infected cells is typically 25–30%.

For stimulation with heat-killed *Salmonella*, bacteria were prepared as described above, except that before infection, bacteria were washed twice in PBS, incubated for 2 h at 80 °C, and then resuspended in complete medium. Treatment was performed under the same conditions as for live bacteria and the cells were collected 20 h post-treatment.

For treatment with bacterial secretome, *Salmonella* was grown in LB medium as described above until OD_600_ 2. Bacterial culture was centrifuged for 2 min at 12,000*g*, the supernatant was harvested, centrifuged for 5 min at 12,000*g* and then the supernatant was filtered through 0.22 µm syringe filters (Millipore, SLGV033RB). Cfu assays confirmed the absence of *Salmonella* in the secretome samples after processing. Treatment was performed in HeLa cells seeded in 24-well plates at a dilution of 1:5 of bacterial supernatant in cell culture medium for 24 h.

To quantify bacterial invasion or intracellular replication by cfu assays, cells were washed three times with PBS and lysed with PBS containing 0.1% Triton-X-100. Cell lysates were serially diluted in PBS and plated on LB (*Salmonella* or *Shigella*) or BHI plates (*Listeria*).

### Secretome collection and cell treatment

For secretome collection, HeLa cells were seeded in six-well plates and were mock-treated or infected 48 h later with *Salmonella* (MOI 100) as described above, except that the medium exchange after antibiotic treatment was done with 2 ml of serum-free medium containing 10 μg/ml gentamicin. At 20 hpi, the secretome was collected and centrifuged for 10 min at 300*g* at 4 °C. The supernatants were filtered through 0.22 µm syringe filters (Millipore, SLGV033RB) and supplemented with 10% fetal bovine serum (Biochrom, S0115). Cfu assays confirmed the absence of *Salmonella* in the secretome samples after processing.

To treat naive cells with the secretome, HeLa cells were seeded at a density of 6.0 × 10^4^ or 2.4 × 10^5^ cells per well in 24- or 6-well plates, respectively, and treated with the secretome 48 h later (500 µl or 2 ml per well in 24- or 6-well plates, respectively). Twenty-four hours after treatment cells were collected and further processed for western blot or RNA isolation as described below.

For *Salmonella* infection of cells treated with secretome, HeLa cells were seeded in 24-well plates, treated with the secretome the following day for 24 h, and then infected with *Salmonella* (MOI 10) as described above.

To digest proteins from the secretome, the secretome collected as described above was treated with Proteinase K-agarose (50 µl/ml of secretome; Sigma, P9290) for 2 h at 37 °C.

To prepare secretome samples for western blot, the secretome was collected and filtered as described above. Two hundred microliters of 100% solution of trichloroacetic acid (Roth, 8789.1) was mixed with 1.8 ml of secretome and incubated for 16 h at 4 °C. Proteins were collected by centrifugation (15,000*g*, 15 min, 4 °C) and washed twice with ice-cold 100% acetone. Pellets were resuspended in Laemmli buffer.

### siRNA transfection

siRNAs were transfected into HeLa cells by a standard reverse transfection protocol, using the transfection reagent Lipofectamine RNAi-MAX (Invitrogen, 13778030). For E2F1 and IRE1 (SMARTpool) siRNA transfection, cells were transfected at a final concentration of 5 nM; for IRE1 (individual siGENOME), JNK1, JNK2, PERK, ATF6, HMGB1, and RAGE siRNAs, cells were transfected at a final concentration of 50 nM.

siGENOME non-targeting siRNA #5 (D-001210-05), SMARTpool siGENOME IRE1 (M-004951-02-0005), individual siGENOME Human IRE1 (D-004951-01, D-004951-02, D-004951-018), SMARTpool siGENOME ATF6 (M-009917-01), SMARTpool siGENOME PERK (M-004883-03), SMARTpool siGENOME JNK1 (M-003514-04), SMARTpool siGENOME JNK2 (M-003505-02), SMARTpool siGENOME HMGB1 (M-018981-01), SMARTpool siGENOME RAGE (M-003625-02-0005), individual siGENOME Human E2F1 siRNA (D-003259-07, D-003259-08) were purchased from Dharmacon. E2F1 (HA04955600-04) predesigned siRNA was purchased from Sigma.

### Cell sorting

HeLa cells were seeded in six-well plates (2.4 × 10^5^ cells per well) and infected 48 h later with *Salmonella* (MOI 100) as described above. At 20 h after infection, cells were trypsinized and collected in PBS. Sorting of the GFP-negative cells (bystander) and GFP-positive (*Salmonella* positive) cells was performed using a FACSAria III flow cytometer (BD Biosciences) based on the signal intensity from the FITC-A channel. Mock-treated cells were also subjected to the same procedure. Sorted cells (3.0 × 10^5^ cells for each fraction) were collected and further processed for western blot and RNA isolation as described below.

### E2F1 overexpression

For the overexpression of E2F1, the coding sequence of E2F1 was excised from HA-E2F-1 wt-pRcCMV (Addgene plasmid # 21667, a gift from William Kaelin) and cloned as a C-terminal fusion of the GFP protein in pEGFP-C1 plasmid (Clontech), in the *Bam*HI restriction site.

1 × 10^6^ HeLa cells were electroporated with 4 µg of pEGFP-E2F1 or pEGFP-C1 (empty vector), using a Gene Pulser Xcell Electroporation System (Biorad), according to the manufacturer’s instructions. After 48 h, cells were infected with *Salmonella* as described above. Cells were collected for western blot or RNA isolation at 20 hpi.

### Protein extracts and western blot

Cells were washed with PBS, lysed in Laemmli’s sample buffer, sonicated, and separated in SDS-PAGE followed by western blotting. For phosphorylated JNK samples, cells were lysed in RIPA buffer containing PhosSTOP (Sigma, 4906845001).

For piglet tissue, intestinal sections of ca. 1 cm^2^ were processed as previously described^81^. Briefly, mucosa scrapings were homogenized in Laemmli’s sample buffer containing Proteinase Inhibitor Cocktail (Sigma-Aldrich, P8340) using pre-chilled mortar and pestles, sonicated, and centrifuged at 10,000*g* for 15 min. Protein samples were separated in SDS-PAGE, followed by western blotting.

The following antibodies were used: β-actin (1:5000; Sigma, A2228, RRID:AB_476697), α-tubulin (1:3000; Sigma, T6074, RRID:AB_477582), E2F1 (1:100; Santacruz, sc-251, RRID:AB_627476), E2F1 (1:1000; Sigma, SAB2103144, RRID:AB_10666369), IRE1 (1:1000; Cell Signaling, 3294, RRID:AB_823545), phosphorylated IRE1 (1:1000; Abcam, ab48187, RRID:AB_873899), BiP (1:1000; Cell Signaling, 3177, RRID:AB_2119845), HMGB1 (1:100; Santacruz, sc-56698, RRID:AB_783817), ASNS (1:100; Santacruz, sc-365809, RRID:AB_10843357), RAGE (1:100; Santacruz, sc-80652, RRID:AB_1128924), PERK (1:500, Santacruz, sc-377400, RRID:AB_2762850), phosphorylated PERK (1:1000; Cell Signaling, 3179, RRID:AB_2095853), ATF6 (1:1000; Abcam, ab122897, RRID:AB_10899171), RTCB (1:1000; proteintech, 19809-1-AP, RRID:AB_10598327), phosphorylated JNK (1:500; Cell Signaling, 4668, RRID:AB_823588), JNK (1:1,000; Cell Signaling, 9252, RRID:AB_2250373), GADPH (1:500; GenScript, A01622-40, RRID:AB_2622160), and anti-mouse and anti-rabbit secondary antibodies coupled to horseradish peroxidase (1:10,000; GE Healthcare, NA931 and NA934, respectively). Signals were detected using SuperSignal West Dura Extended Duration Substrate (Pierce, 34075) using an ImageQuant LAS 4000 CCD camera (GE Healthcare).

Of note, long-term treatment with tunicamycin results in a blockade of N-linked glycosylation, which results in a fully unglycosylated ATF6 detectable as a faster migrating form of ATF6 (approx. 100 kDa). This unglycosylated ATF6 form cannot undergo proteolytic processing into the active ATF6 transcription factor^82,83^.

Quantification of western blots was performed with ImageJ. Uncropped images of immunoblots are included as Source Data.

### Sample preparation, mass spectrometry data acquisition, and analysis

Twenty-two milliliters of cell culture medium from HeLa cells mock-treated or infected with *Salmonella* (MOI 100) were collected at 14 hpi and processed as described above. Secretomes were concentrated by centrifugation using Amicon filters with a 3 kDa cut-off value (Merk-Millipore, UFC900308). Samples from four independent experiments were collected.

Samples were processed for mass spectrometry at the EMBL Proteomics Core Facility. Disulfide bonds were reduced by adding 10 mM final concentration of DTT in 50 mM HEPES, pH 8.5, and incubating the samples for 30 min at 56 °C. Subsequently, an alkylation step was performed by adding 20 mM 2-chloroacetamide in 50 mM HEPES, pH 8.5, and incubating the samples in dark for 30 min at RT. Four washes with 50 mM HEPES were performed in Amicon filter tubes (3 kDa cut-off) to concentrate the sample, remove the phenol red, and exchange the sample buffer to optimal digestion conditions. For digestion, volume was adjusted to 100 µL with 50 mM HEPES and trypsin (Promega, V5111) was added at a 1:25 enzyme to protein ratio for overnight digestion at 37 °C. Peptides were eluted by centrifugation (10 min, 12,000*g*), followed by a second elution with 100 µL of 50 mM HEPES. Peptides were labeled with TMT10plex Isobaric Label Reagent (ThermoFisher, 90110)^84^, according to the manufacturer’s instructions. For further sample clean-up an OASIS HLB µElution Plate (Waters, 186001828BA) was used. Replicates were subjected to offline high pH reverse phase fractionation, carried out on an Agilent 1200 Infinity HPLC system, equipped with a Gemini C18 column, resulting in six fractions^85^.

Peptides were separated using the UltiMate 3000 RSLC nano LC system (Dionex) fitted with a trapping cartridge (µ-Precolumn C18 PepMap 100, 5 µm, 300 µm i.d. × 5 mm, 100 Å) and an analytical column (nanoEase™ M/Z HSS T3 column 75 µm × 250 mm C18, 1.8 µm, 100 Å, Waters). Trapping was carried out with a constant flow of solvent A (0.1% formic acid in water) at 30 µL/min onto the trapping column for 6 min. Subsequently, peptides were eluted via the analytical column with a constant flow of 0.3 µl/min with an increasing percentage of solvent B (0.1% formic acid in acetonitrile) from 2 to 4% in 4 min, from 4 to 8% in 2 min, then 8 to 28% for a further 96 min, and finally from 28 to 40% in another 10 min. The outlet of the analytical column was coupled directly to a QExactive plus (Thermo) mass spectrometer using the proxeon nanoflow source in positive ion mode. The peptides were introduced into the mass spectrometer QExactive plus via a Pico-Tip Emitter 360 µm OD × 20 µm ID; 10 µm tip (New Objective) and a spray voltage of 2.3 kV was applied. The capillary temperature was set at 320 °C. Full mass scan was acquired with mass range 350–1400*m*/*z* in profile mode in the FT with a resolution of 70,000. The filling time was set at a maximum of 100 ms with a limitation of 3 × 10^6^ ions. Data-dependent acquisition was performed with the resolution of the Orbitrap set to 35,000, with a fill time of 120 ms and a limitation of 2 × 10^5^ ions. A normalized collision energy of 32 was applied. A loop count of 10 with count 1 was used and a minimum AGC trigger of 2e^2^ was set. Dynamic exclusion time of 30 s was used. The peptide match algorithm was set to “preferred” and charge exclusion “unassigned”, charge states 1, 5–8 were excluded. MS2 data were acquired in profile mode^86^.

IsobarQuant^87^ and Mascot (v2.2.07; Matrix Science, London, UK) were used to process the acquired data, which was searched against the Uniprot Homo sapiens proteome database (UP000005640) containing common contaminants and reversed sequences. The following modifications were included into the search parameters: Carbamidomethyl (C) and TMT10 (K) (fixed modification), Acetyl (N-term), Oxidation (M), and TMT10 (N-term) (variable modifications). For the full scan (MS1) a mass error tolerance of 10 ppm and for MS/MS (MS2) spectra of 0.02 Da was set. Further parameters were set as follows: trypsin as a protease with an allowance of maximum two missed cleavages; a minimum peptide length of seven amino acids; at least two unique peptides were required for protein identification. The false discovery rate on peptide and protein level was set to 0.01.

The raw output files of IsobarQuant (protein.txt—files) were processed using the R programming language. Only proteins that were quantified with at least two unique peptides were considered for the analysis. Moreover, only proteins that were identified in two out of two mass spec runs were kept. Nine hundred and forty-eight proteins passed the quality control filters. Raw signal-sums (signal_sum columns) were first cleaned for batch effects using the “removeBatchEffect” function of the limma package (v. v3.34.5)^88^ and further normalized using vsn (variance stabilization normalization^89^). The four replicates of the HeLa wild-type condition were normalized separately to maintain the higher protein abundance in this condition. Proteins were tested for differential expression using the limma package. A protein was annotated as a hit with a false discovery rate (fdr) smaller 5% and a fold change of at least 100% and as a candidate with an fdr below 20% and a fold change of at least 50%.

Functional analysis of the proteins significantly increased in the secretome of *Salmonella-*infected cells compared to mock-treated cells (722 proteins) was conducted using the Ingenuity Pathway Analysis Software (IPA, Ingenuity Systems). Datasets were uploaded into IPA and analyzed for functional enrichment in terms of “Canonical pathways”, based on the information in the Ingenuity Knowledge Base. Enrichments were calculated by IPA using multiple hypothesis correction based on the Benjamini–Hochberg method; corrected *P* values are shown.

The mass spectrometry proteomics data have been deposited to the ProteomeXchange Consortium via the PRIDE^90^ partner repository with the dataset identifier PXD018026 (http://www.ebi.ac.uk/pride/archive/projects/PXD018026).

### Fluorescence staining and confocal microscopy

Cells seeded in glass coverslips in 24-well plates and treated as described above were fixed with 4% paraformaldehyde (PFA) for 15 min at RT, permeabilized with 0.5% Triton-X-100 in PBS for 10 min. Nuclei were counterstained with Hoechst 33342 (1:5,000; Life Technologies, H3570). Slides were mounted in Vectashield (VectorLabs, H-1000).

Confocal microscopy images, shown as maximum projected *Z*-stack images, were acquired with a Leica SP5 laser scanning confocal microscope (Leica Microsystems).

### RNA isolation, PCR, and quantitative real-time PCR

For total RNA isolation, including small RNA fraction, cells were lysed in TRIzol (Invitrogen, 15596026) and RNA was extracted by phenol–chloroform followed by isopropanol precipitation. For the piglet ileum and colon tissue, total RNA was extracted using mirVana miRNA Isolation kit (Invitrogen, AM1561) according to the manufacturer’s instructions.

For quantification of gene expression, total RNA was reverse transcribed using hexameric random primers and M-MLV reverse transcriptase (Invitrogen, 28025021), according to the manufacturer’s instructions. qRT-PCR was performed using SsoAdvanced Universal SYBR Green Supermix (BioRad, 172-5274) according to the manufacturer’s instructions. The primer pairs used are indicated in Supplementary Table 1. Expression was normalized to β-actin or RPL37a.

For the XBP1 splicing assay, PCR was performed using *Taq* DNA polymerase (NEB, M0273), according to the manufacturer’s instructions. The primer pairs used are indicated in Supplementary Table 1. Amplified fragments covering the XBP1 intron and flanking exon fragments were separated on 6% polyacrylamide gels.

For mature miRNA quantification, RNA was reverse transcribed using the miRCURY LNA Universal cDNA synthesis kit (Qiagen, 339340) followed by qRT-PCR using predesigned miRCURY LNA PCR primer sets (Qiagen) and miRCURY LNA SYBR Green master mix (Qiagen, 339347), according to the manufacturer’s instructions. The following primer sets, for both human and piglet miRNAs, were used: miR-15a-5p (Qiagen, YP00204066), miR-15b-5p (Qiagen, YP00204243), miR-16-5p (Qiagen, YP00205702), miR-22-3p (Qiagen, YP00204606), miR-421 (Qiagen, YP00204603), miR-744-5p (Qiagen, YP00204663), let-7i-3p (Qiagen, YP00204247), let-7i-5p (Qiagen, YP00204394), miR-29a-3p (Qiagen, YP00204698). Expression was normalized to miR-29a-3p.

qRT-PCR was performed using a CFX96 TouchTM Real-Time PCR detection system (BioRad). Relative gene expression was calculated using the 2^−ΔΔCt^ method.

### RNA sequencing and computational RNA-seq analysis

Library preparation and deep-sequencing were performed by the EMBL Genomics Core Facility. Briefly, RNA integrity was checked using the RNA Nano 6000 Assay Kit (Agilent Technologies, 5067-1511) in a Bioanalyzer 2100 system (Agilent Technologies), and concentration was measured with Qubit RNA Assay Kit (Invitrogen, Q10211) in a Qubit 2.0 Fluorometer (Invitrogen). Small RNA-seq libraries were prepared manually from 250 ng of total RNA using the NEBNext Multiplex Small RNA Library Prep kit (New England Biolabs, E7300L). Obtained libraries that passed the QC step, which was assessed on the Agilent Bioanalyzer system, were pooled in equimolar amounts. Ten picomolar solution of each pool of libraries was loaded on the Illumina sequencer HiSeq2500 and sequenced uni-directionally, generating ~190 million reads per run, each 62 bases long.

For data analysis, the quality of the reads was analyzed using FastQC (version 0.11.8). The reads were trimmed and adapter sequences were removed using Trimmomatic (version 0.38)^91^. The reads in fastq format were converted to fasta format using fastq_to_fasta commands in the FASTX Toolkit (version 0.0.13). Further read processing, including polyA-tail removal, size filtering (minimal length 12 nt. after clipping), alignment of the reads, computation of alignment statistics, and annotation, was performed using the READemption pipeline (version 0.4.3)^92^ with default parameters, with Segemehl (version 0.2.0)^93^. Reads were mapped against the human mature miRNA sequences provided by miRBase (release 22). For normalization, read counts of each miRNA species were normalized by those of miR-29a-3p (a miRNA that does not change with *Salmonella* infection or E2F1 knockdown). This normalization strategy has been previously used for the normalization of small RNA-seq datasets in which global changes of miRNA expression are observed^94^.

The demultiplexed FASTQ files and coverage files have been deposited in NCBI’s Gene Expression Omnibus and are accessible through GEO Series accession numbers GSE147362 (*Salmonella*-infected cells), GSE147361 (E2F1 knockdown samples), and GSE147363 (cells treated with secretome).

### Piglet infections and sample preparation

*Salmonella* infection of piglets and sample preparation were performed as described previously^28,95^. Briefly, six piglets were challenged orally with 10^8^ cfu of *Salmonella* Typhimurium, whereas three piglets (mock group) received sterile medium. The mock group was necropsied prior to the infection group and subsequently, three animals were necropsied at 2 or 6 days post-infection (dpi). Sections of mucosal tissue from the ileum and colon were independently sectioned in pieces of 10 cm^2^ and immediately frozen in liquid nitrogen for protein and RNA isolation.

All procedures involving animals were performed at the animal facility of the University of Leon, Spain, and were approved by the institutional bioethical committee of the University of Leon, Spain (license number ULE_003_2005, approval date 25 January 2005) and performed according to European regulations regarding animal welfare and protection of animals used for experimental and other scientific purposes. Statistical consideration was not used to determine the animal sample size. Animals were randomly assigned to experimental groups; data blinding was not performed.

### Statistical analysis

Unless otherwise indicated, data are presented as mean ± standard error of the mean (s.e.m.), with the exact number of experiments performed indicated in figure legends. Statistical analysis was performed using Prism Software (GraphPad). Normal distribution of the data was assessed by the Shapiro–Wilk test. For statistical comparison of datasets from two conditions, two-tailed Student’s *t*-test or multiple *t*-test corrected for multiple comparison using the Holm–Sidak method were used; for data from three or more conditions/groups, one-way ANOVA with Tukey’s or Dunnett’s post hoc test or two-way ANOVA with Tukey’s multiple comparison test was used. For non-parametric data, Wilcoxon signed rank or Kruskal–Wallis with Dunn’s multiple comparison tests were used. Values of *P* < 0.05 were considered significant. Heatmaps were plotted using TIBCO Spotfire software. Statistical analyses are detailed in Supplementary Data 1.

### Reporting summary

Further information on research design is available in the Nature Research Reporting Summary linked to this article.

## Supplementary information

Supplementary Information

Peer Review File

Description of Additional Supplementary Files

Supplementary Data 1

Supplementary Data 2

Supplementary Data 3

Reporting Summary

## Data Availability

The data supporting the findings of this study are available from the corresponding author upon reasonable request. The mass spectrometry proteomics data have been deposited to the ProteomeXchange Consortium via the PRIDE partner repository with the dataset identifier PXD018026. The demultiplexed FASTQ files and coverage files have been deposited in NCBI’s Gene Expression Omnibus and are accessible through GEO Series accession numbers GSE147362 (*Salmonella*-infected cells), GSE147361 (E2F1 knockdown samples), and GSE147363 (cells treated with secretome). The Uniprot Homo sapiens proteome database (UP000005640) can be accessed at https://www.uniprot.org/proteomes/UP000005640. The human mature miRNA sequences can be accessed at http://www.mirbase.org/. Source data are provided with this paper.

## Source data

Source Data
