# Supplementary material for: Reprogramming of microRNA expression via E2F1 downregulation promotes Salmonella infection both in infected and bystander cells
Source: Nat Commun. 2021 Jun 7;12:3392. doi: 10.1038/s41467-021-23593-z (PMC8184997; doi:10.1038/s41467-021-23593-z)
Supplement: Supplementary file 1 — Supplementary Information [file 41467_2021_23593_MOESM1_ESM.pdf]

SUPPLEMENTARY INFORMATION

Reprogramming of microRNA expression via E2F1 downregulation promotes *Salmonella* infection both in infected and bystander cells

Carmen Aguilar, Susana Costa, Claire Maudet, R.P. Vivek-Ananth, Sara Zaldívar-López, Juan J. Garrido, Areejit Samal, Miguel Mano, Ana Eulalio

Corresponding author:
Ana Eulalio; e-mail: aeulalio@ci.uc.pt ; ORCID: 0000-0002-7355-0674

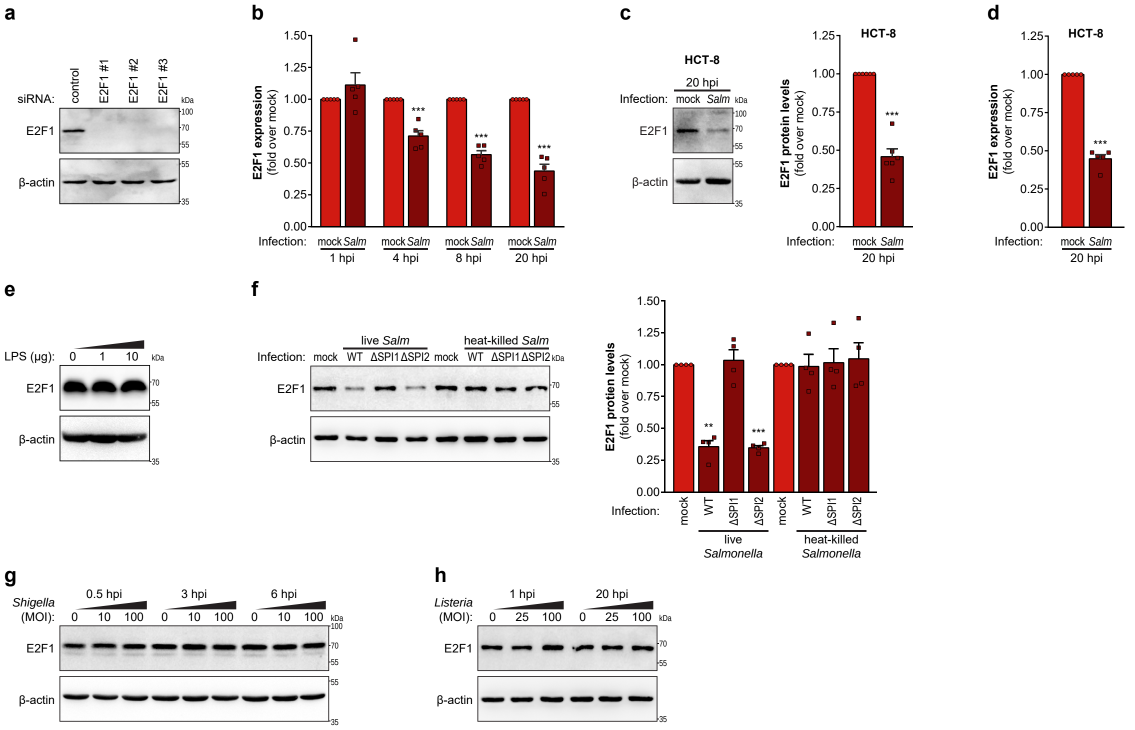

Supplementary Figure 1. E2F1 expression is not changed by extracellular *Salmonella* stimuli

a. E2F1 protein levels, determined by Western-blot, in HeLa cells transfected with control or 3 independent siRNAs targeting E2F1.
b. E2F1 expression levels, determined by qRT-PCR, in HeLa cells infected with *Salmonella* or mock-treated, analyzed at 1, 4, 8, and 20 hpi.
c and d. E2F1 expression levels, determined by Western-blot (c) or qRT-PCR (d), in HCT-8 cells infected with *Salmonella* or mock-treated, analyzed at 20 hpi.
e. E2F1 protein levels, determined by Western-blot, upon treatment with purified *Salmonella* LPS for 24 h.
f. E2F1 protein levels, determined by Western-blot, in HeLa cells infected with live or treated with heat-killed *Salmonella* wild-type (WT), ΔSPI-1 (invasion defective) or ΔSPI-2 (defective in intracellular replication) mutant strains, analyzed at 20 hpi.
g. E2F1 protein levels, determined by Western-blot, in HeLa cells infected with *Shigella flexneri* (MOI 10 and 100) or mock-treated, analyzed at 0.5, 3, and 6 hpi.
h. E2F1 protein levels, determined by Western-blot, in HeLa cells infected with *Listeria monocytogenes* (MOI 25 and 100) or mock-treated, analyzed at 1 and 20 hpi.
Salmonella infection was performed at MOI 100. Results are shown as mean±s.e.m. of n=4 (f), n=5 (b, d) or n=6 (c) biologically independent experiments; Western-blots are representative of n=3 (a, e, g, h), n=4 (f) or n=6 (c) biologically independent experiments; **P<0.01 and ***P<0.001 (statistical analysis is detailed in Supplementary Data 1); Source data are provided as a Source Data file.

Aguilar et al. - Supplementary Figure 2

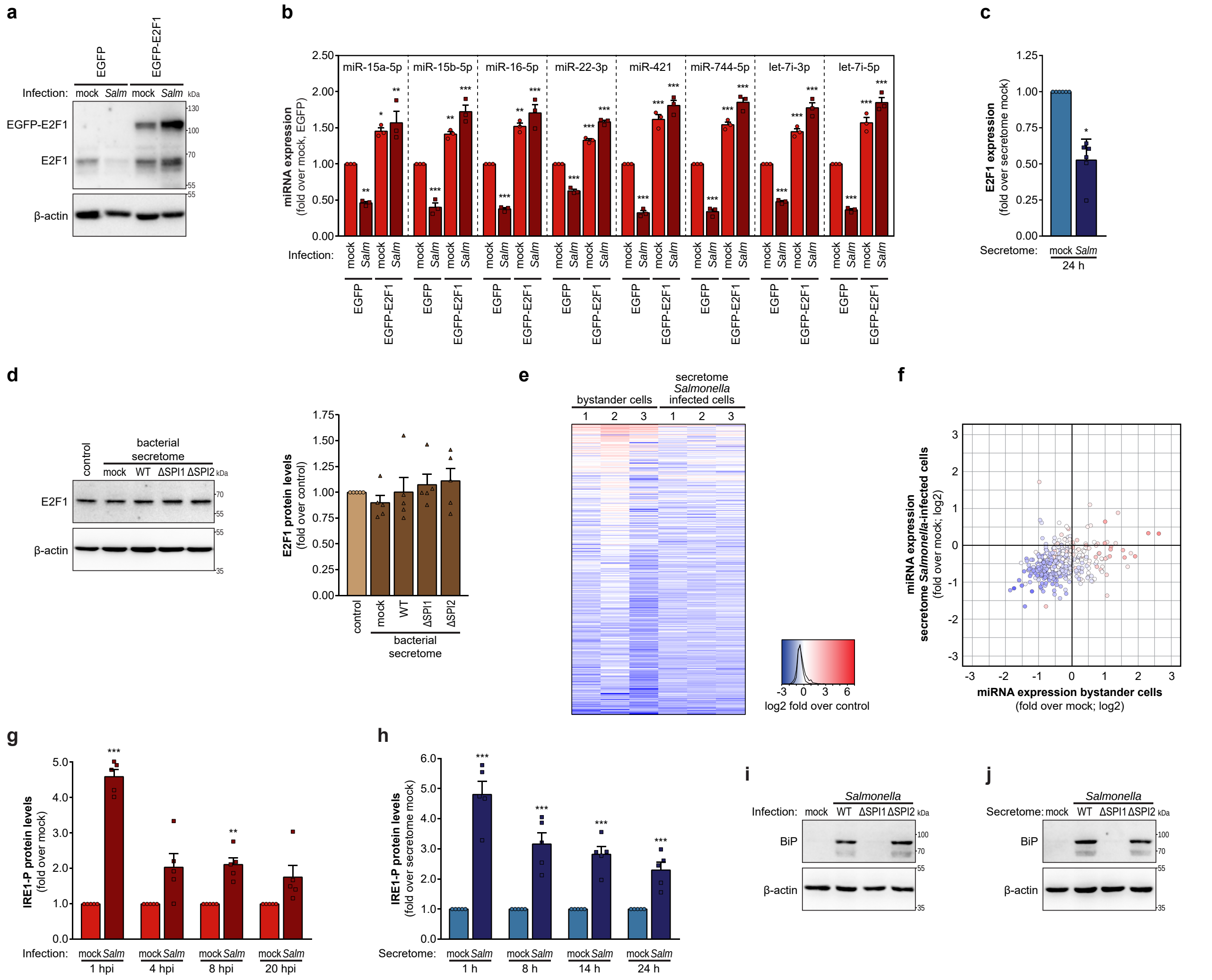

Supplementary Figure 2. *Salmonella* secretome has no effect on E2F1 expression

a. E2F1 expression, determined by Western-blot, in HeLa cells expressing EGFP or EGFP-E2F1, and infected with *Salmonella* or mock-treated, analyzed at 20 hpi.

b. Expression levels of the mature forms of selected miRNAs determined by qRT-PCR in HeLa cells expressing EGFP or EGFP-E2F1, and infected with *Salmonella* or mock-treated, analyzed at 20 hpi. Results are normalized to cells expressing EGFP and mock-treated.

c. E2F1 expression, determined by qRT-PCR, in naïve HeLa cells treated with the secretome of *Salmonella*-infected or mock-treated cells. Results are normalized to naïve cells treated with the secretome collected from mock-treated cells.

d. E2F1 protein levels, determined by Western-blot, in HeLa cells treated with the secretome of the indicated *Salmonella* strains or mock-treated for 24 h.

e. Heat map showing miRNA expression changes of bystander cells (same as Fig. 1g) and naïve HeLa cells treated with the secretome of *Salmonella*-infected cells (24 post-treatment; same as Fig. 2c). Results are shown as log2 fold change compared to mock-treated cells, or to naïve cells treated with secretome collected from mock-treated cells, respectively. RNA-seq datasets from 3 independent experiments are shown. Only miRNAs with a number of reads ≥20 in both controls are shown

f. Comparison of miRNA expression changes in bystander cells and naïve HeLa cells treated with the secretome of *Salmonella*-infected cells. Color code for datapoints identical to panel e, based on average values.

g and h. Quantification of IRE phosphorylation, based on Western-blot, in HeLa cells either infected with *Salmonella* or mock-treated (g) or treated with the secretome of *Salmonella*-infected cells or mock-treated cells (h). Representative Western-blots are shown in Fig. 2j and 2n, respectively.

i and j. BiP protein levels, determined by Western-blot, in HeLa cells infected (i) or treated with the secretome of cells (j) infected with *Salmonella* wild-type (WT), ΔSPI-1 (invasion defective) or ΔSPI-2 (defective in intracellular replication) mutant strains, analyzed at 20 hpi or 24 h post-treatment, respectively.

Salmonella infection was performed at MOI 100. Results are shown as mean±s.e.m. of n=3 (b) or n=5 (c, d, g, h) biologically independent experiments. Western-blots are representative of n=3 (i, j) or n=5 (a, d) biologically independent experiments; *P<0.05, **P<0.01 and ***P<0.001 (statistical analysis is detailed in Supplementary Data 1); Source data are provided as a Source Data file.

Aguilar et al. - Supplementary Figure 3

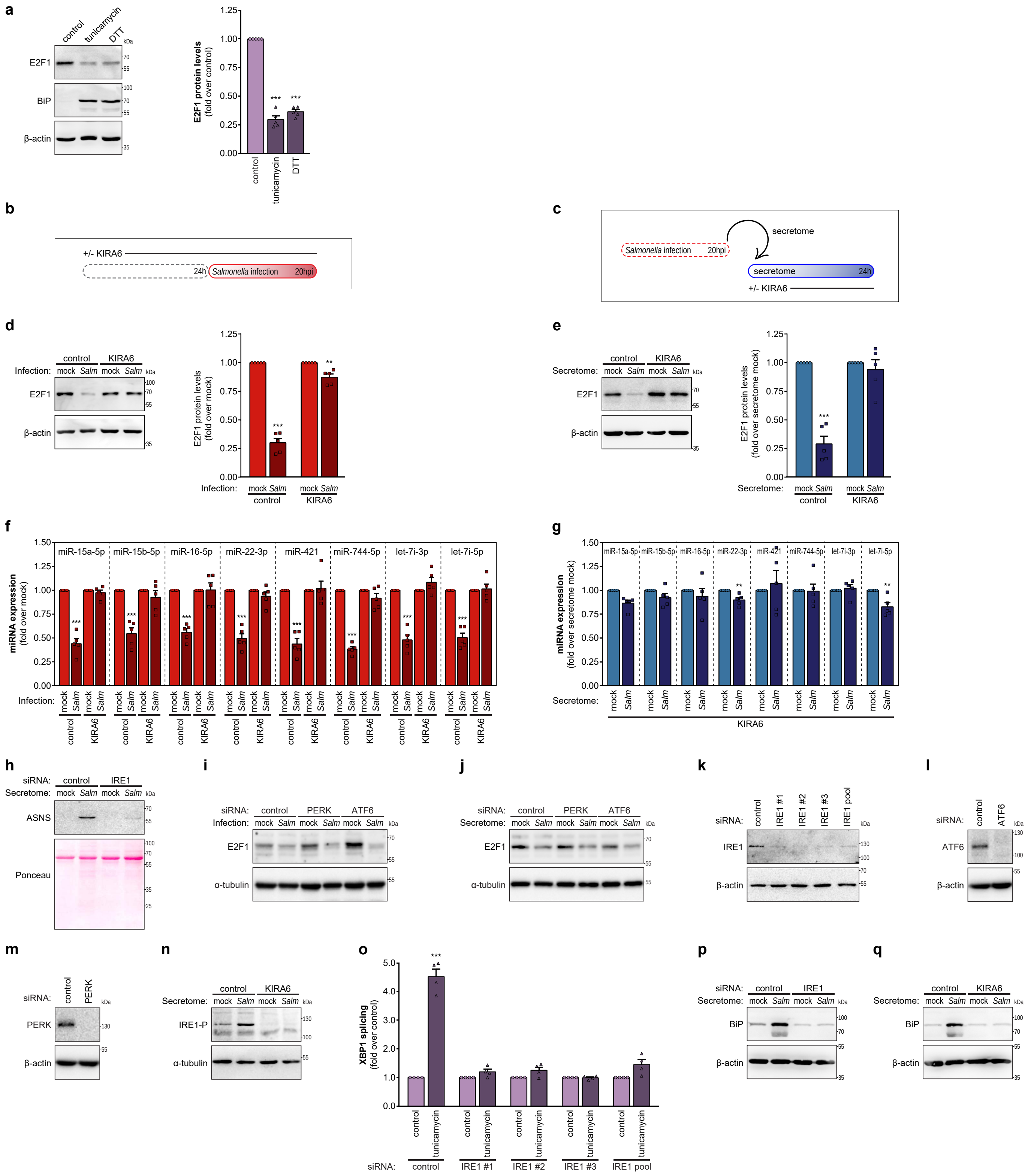

Supplementary Figure 3. Inhibition of IRE1 prevents E2F1 downregulation triggered by *Salmonella* infection or by the secretome of infected cells

a. E2F1 and BiP protein levels, determined by Western-blot, upon treatment of HeLa cells with DTT (1 mM, 24 h) or tunicamycin (0.5 μg/ml, 24 h).

b. Schematic representation of the experimental design for the infection experiments. Cells were pre-treated with control (DMSO) or the IRE1 kinase activity inhibitor KIRA6 for 24 h, and then cells were infected with *Salmonella*. The inhibitor/control treatment was maintained during infection. Samples were collected at 20 hpi.

c. Schematic representation of the experimental design for the secretome experiments. Cells were treated with the secretome of *Salmonella*-infected cells for 24 h, in the absence (control, DMSO) or presence of KIRA6. The secretome of mock-treated cells was used for comparison.

d and e. E2F1 protein levels, determined by Western-blot, in HeLa cells treated with KIRA6 or control, and either infected with *Salmonella* or mock-treated (d) or treated with the secretome of *Salmonella*-infected cells or mock-treated cells (e).

f. Expression levels of selected miRNAs, determined by qRT-PCR, in HeLa cells treated with KIRA6 or control, and infected with *Salmonella* or mock-treated, analyzed at 20 hpi. Results are normalized to mock-treated cells.

g. Expression levels of selected miRNAs, determined by qRT-PCR, in HeLa treated the secretome of *Salmonella*-infected cells or mock-treated cells for 24 h, in the presence of KIRA6 or vehicle. Results are normalized to naïve cells treated with secretome collected from mock-treated cells.

h. ASNS protein levels, determined by Western-blot, in the secretome of HeLa cells transfected with control or IRE1 siRNAs and infected with *Salmonella* or mock-treated. Ponceau staining of the membranes is shown.
i and j. E2F1 protein levels, determined by Western-blot, in HeLa cells transfected with control, PERK or ATF6 siRNAs, and either infected with *Salmonella* or mock-treated (20 hpi; i) or treated with the secretome of *Salmonella*-infected cells or mock-treated cells (24 h; j).
k. IRE1 protein levels, determined by Western-blot, in HeLa cells transfected with control, 3 independent siRNAs or a pool of siRNAs targeting IRE1.
l and m. ATF6 and PERK protein levels, determined by Western-blot, in HeLa cells either transfected with control or ATF6 siRNAs (l) or control or PERK siRNAs (m).
n. IRE phosphorylation, determined by Western-blot, in HeLa cells treated with control or KIRA6 and treated with the secretome of *Salmonella*-infected or mock-treated cells for 1 h.
o. Expression levels of XBP1s, determined by qRT-PCR, in HeLa cells transfected with control, 3 independent siRNAs or a pool of siRNAs targeting IRE1, and treated with control (DMSO) or tunicamycin (2 µg/ml, 6 h). Results are normalized to control-treated cells.
p and q. BiP protein levels, determined by Western-blot, in HeLa cells transfected with control or IRE1 siRNAs (p) or treated with control or KIRA6 (q) and treated with the secretome of *Salmonella*-infected cells or mock-treated cells for 24 h.
Salmonella infection was performed at MOI 100. Results are shown as mean±s.e.m. of n=4 (o) or n=5 (a, d, e, f, g) biologically independent experiments; Western-blots are representative of n=3 (h, i, j, k, l, m, n) or n=5 (a, d, e, p, q) biologically independent experiments; **P<0.01 and ***P<0.001 (statistical analysis is detailed in Supplementary Data 1); Source data are provided as a Source Data file.

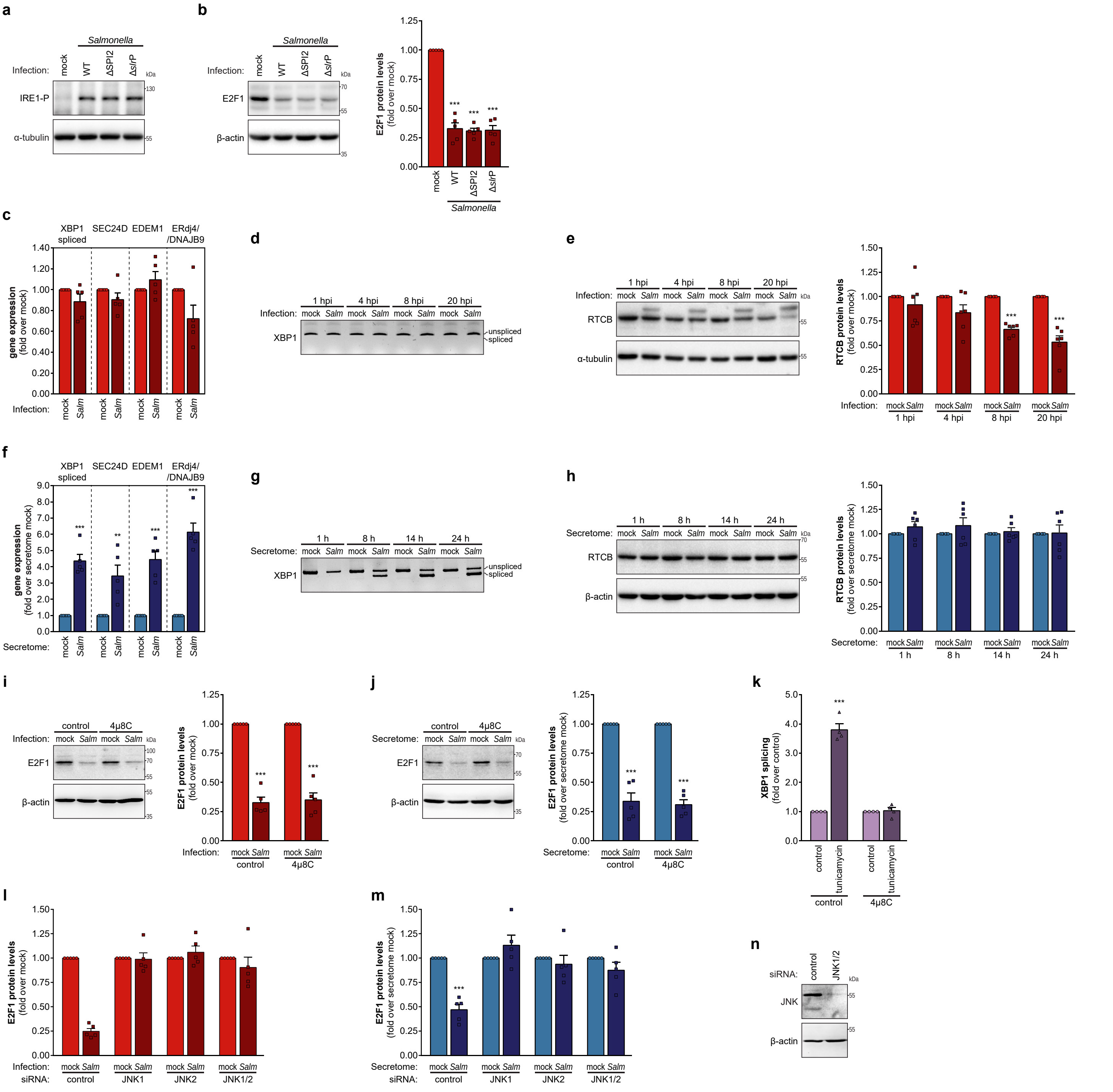

Supplementary Figure 4. *Salmonella* effector protein SlrP is not the trigger for ER-stress activation upon infection

a and b. IRE1 phosphorylation (A) and E2F1 protein levels (B), determined by Western-blot, in HeLa cells mock-treated or infected with *Salmonella* wild-type (WT), ΔSPI-2 or ΔslrP mutant strains, analyzed at 1 or 20 hpi, respectively.

c and f. Expression levels of spliced XBP1 (XBP1s), and XBP1s downstream targets (SEC24D, EDEM1, ERdj4/DNAJB9), determined by qRT-PCR, in HeLa cells either infected with *Salmonella* or mock-treated (20 hpi; c) or treated with the secretome of *Salmonella*-infected cells or mock-treated cells (24 h; f). Results are normalized to mock-treated cells.

d and g. Splicing of XBP1 mRNA detected by electrophoresis after RT-PCR, in HeLa cells either infected with *Salmonella* or mock-treated (d) or treated with the secretome of *Salmonella*-infected cells or mock-treated cells (g).

e and h. Levels of RTCB protein, determined by Western-blot, in HeLa cells either infected with *Salmonella* or mock-treated (e) or treated with the secretome of *Salmonella*-infected cells or mock-treated cells (h).

i and j. E2F1 protein levels, determined by Western-blot, in HeLa cells treated with the IRE1 endonuclease activity inhibitor 4μ8C or control (DMSO), and either infected with *Salmonella* or mock-treated (20 hpi; i) or treated with the secretome of *Salmonella*-infected cells or mock-treated cells (24 h; j).

k. Expression levels of XBP1s, determined by qRT-PCR, in HeLa cells pre-treated with 4μ8C (1 h, 50 μM) and then simultaneously treated with 4μ8C (25 μM) and tunicamycin (2 μg/ml) for 6 h. Results are normalized to control-treated cells.

l and m. Quantification of E2F1 protein levels, determined by Western-blot, in HeLa cells transfected with control, JNK1, JNK2 or JNK1/JNK2 siRNAs, and either infected with *Salmonella* or mock-treated (20 hpi; l) or treated with the secretome of *Salmonella*-infected cells or mock-treated cells (24 h; m). Representative Western-blots are shown in Fig. 3h and 3j, respectively.

n. JNK protein levels, determined by Western-blot, in HeLa cells transfected with control or JNK1/JNK2 siRNAs.

Salmonella infection was performed at MOI 100. Results are shown as mean±s.e.m. of n=3 (k), n=5 (b, c, f, i, j, l, m) or n=6 (e, h) biologically independent experiments; Western-blots are representative of n=3 (a, n), n=5 (b, i, j) or n=6 (e, h) biologically independent experiments; ***P<0.001 (statistical analysis is detailed in Supplementary Data 1); Source data are provided as a Source Data file.

Aguilar et al. - Supplementary Figure 5

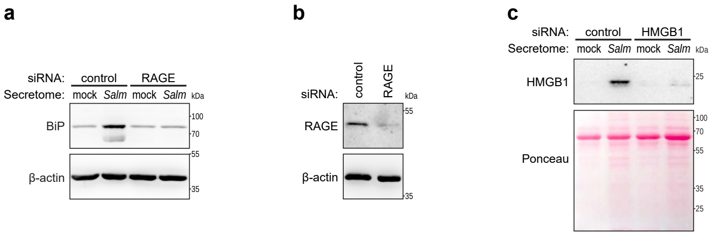

Supplementary Figure 5. Activation of ER-stress response pathway by the secretome of *Salmonella*-infected cells is RAGE-dependent

a. BiP protein levels, determined by Western-blot, in HeLa cells transfected with control or RAGE siRNAs and treated with the secretome of *Salmonella*-infected or mock-treated cells for 24 h.

b. RAGE protein levels, determined by Western-blot, in HeLa cells transfected with control or RAGE siRNAs.

c. HMGB1 protein levels, determined by Western-blot, in the secretome of HeLa cells transfected with control or HMGB1 siRNAs and infected with *Salmonella* or mock-treated. Ponceau staining of the membranes is shown.

Salmonella infection was performed at MOI 100. Western-blots are representative of n=3 (b, c) or n=5 (a) biologically independent experiments; Source data are provided as a Source Data file.

Supplementary Table 1. Primers used in this study.

**Primer**	**Sequence (5'->3')**
qRT-PCR	
RPL37a forward	AAGACTGAAGGAGTTGAA
RPL37a reverse	TTTGTTACATAAATTAACCCATT
β-actin forward	CCTGTACGCCAACACAGTGC
β-actin reverse	ATACTCCTGCTTGCTGATCC
E2F1 forward	GCAGAGCAGATGGTTATG
E2F1 reverse	TGAAAGTTCTCCGAAGAGT
spliced XBP1 (XBP1s) forward	TGGCCGGGTCCAGTTGTCA
spliced XBP1 (XBP1s) reverse	GAACATGACTGGGTCCAAGTTG
ERdj4/DNAJB9 forward	TCTTAGGTGTGCCAAAATCGG
ERdj4/DNAJB9 reverse	TGTCAGGGTGGTACTTCATGG
SEC24D forward	GTAAGCAGCCCACCAGAACT
SEC24D reverse	TTTCCCACTTCAGGCAGCAA
EDEM1 forward	CGGACGAGTACGAGAAGCG
EDEM1 reverse	CGTAGCCAAAGACGAACATGC
XBP1 splicing assay	
XBP1 forward	CCTTGTAGTTGAGAACCAGGAG
XBP1 reverse	GGTCCAAGTTGTCCAGAATGC
