# Supplementary material for: Reprogramming of microRNA expression via E2F1 downregulation promotes Salmonella infection both in infected and bystander cells
Source: Nat Commun. 2021 Jun 7;12:3392. doi: 10.1038/s41467-021-23593-z (PMC8184997; doi:10.1038/s41467-021-23593-z)
Supplement: Supplementary file 3 — Description of Additional Supplementary Files [file 41467_2021_23593_MOESM3_ESM.pdf]

**Description of Additional Supplementary Files**

File Name: Supplementary Data 1
Description: Statistical analyses for data presented in main and supplementary Figures.

File Name: Supplementary Data 2
Description: List of proteins present in the secretome of *Salmonella*-infected cells, identified by mass-spectrometry. Significantly enriched/depleted proteins in the secretome of *Salmonella*-infected cells compared to mock treated cells are classified as hits.

File Name: Supplementary Data 3
Description: Functional categories enriched among the proteins significantly increased in the secretome of *Salmonella* infected cells. Enrichment analysis was performed using IPA, applying Benjamini– Hochberg multiple testing correction; all the enriched categories with a P1.3) are shown.
