# Supplementary material for: Reprogramming of microRNA expression via E2F1 downregulation promotes Salmonella infection both in infected and bystander cells
Source: Nat Commun. 2021 Jun 7;12:3392. doi: 10.1038/s41467-021-23593-z (PMC8184997; doi:10.1038/s41467-021-23593-z)
Supplement: Supplementary file 7 — Reporting Summary [file 41467_2021_23593_MOESM7_ESM.pdf]

nature research

Corresponding author(s): Ana Eulalio
Last updated by author(s): Apr 16, 2021

Reporting Summary

Nature Research wishes to improve the reproducibility of the work that we publish. This form provides structure for consistency and transparency in reporting. For further information on Nature Research policies, see our Editorial Policies and the Editorial Policy Checklist.

Statistics

For all statistical analyses, confirm that the following items are present in the figure legend, table legend, main text, or Methods section.

n/a	Confirmed	
☐	☒	The exact sample size (*n*) for each experimental group/condition, given as a discrete number and unit of measurement
☐	☒	A statement on whether measurements were taken from distinct samples or whether the same sample was measured repeatedly
☐	☒	The statistical test(s) used AND whether they are one- or two-sided<br>*Only common tests should be described solely by name; describe more complex techniques in the Methods section.*
☒	☐	A description of all covariates tested
☐	☒	A description of any assumptions or corrections, such as tests of normality and adjustment for multiple comparisons
☐	☒	A full description of the statistical parameters including central tendency (e.g. means) or other basic estimates (e.g. regression coefficient) AND variation (e.g. standard deviation) or associated estimates of uncertainty (e.g. confidence intervals)
☐	☒	For null hypothesis testing, the test statistic (e.g. *F*, *t*, *r*) with confidence intervals, effect sizes, degrees of freedom and *P* value noted<br>*Give P values as exact values whenever suitable.*
☒	☐	For Bayesian analysis, information on the choice of priors and Markov chain Monte Carlo settings
☒	☐	For hierarchical and complex designs, identification of the appropriate level for tests and full reporting of outcomes
☒	☐	Estimates of effect sizes (e.g. Cohen's *d*, Pearson's *r*), indicating how they were calculated

Our web collection on statistics for biologists contains articles on many of the points above.

Software and code

Policy information about availability of computer code

Data collection	Leica Application Suite Advance Fluorescence (LAS AF; Leica Microsystems, v.2.7.3.9723) , BD FACSDiva software (BD Biosciences, v8.0.1)
Data analysis	Microsoft Excel (Microsoft Office, 2013), Prism (GraphPad, v.7.00), Adobe Photoshop (Adobe, CS6), ImageJ (v.1.50b), IsobarQuant, Mascot (Matrix Science, V.2.2.07), limma software package, (v.3.34.5), FastQC (v.0.11.8), Trimmomatic (v.0.38), Segemehl (v.0.2.0), , READemption pipeline (v 0.4.3), TIBCO Sportfire Analyst (TIBCO, v.7.11.2 LTS), Ingenuity Pathway Analysis Software (IPA, Ingenuity Systems)

For manuscripts utilizing custom algorithms or software that are central to the research but not yet described in published literature, software must be made available to editors and reviewers. We strongly encourage code deposition in a community repository (e.g. GitHub). See the Nature Research guidelines for submitting code & software for further information.

Data

Policy information about availability of data

All manuscripts must include a data availability statement. This statement should provide the following information, where applicable:

- Accession codes, unique identifiers, or web links for publicly available datasets
- A list of figures that have associated raw data
- A description of any restrictions on data availability

The data supporting the findings of this study are available from the corresponding author upon reasonable request. The mass spectrometry proteomics data have been deposited to the ProteomeXchange Consortium via the PRIDE partner repository with the dataset identifier PXD018026 (http://www.ebi.ac.uk/pride/archive/projects/PXD018026). The demultiplexed FASTQ files and coverage files have been deposited in NCBI's Gene Expression Omnibus and are accessible through GEO Series accession numbers GSE147362 (Salmonella-infected cells; https://www.ncbi.nlm.nih.gov/geo/query/acc.cgi?acc=GSE147362), GSE147361 (E2F1 knockdown samples; https://www.ncbi.nlm.nih.gov/geo/query/acc.cgi?acc=GSE147361), and GSE147363 (cells treated with secretome; https://www.ncbi.nlm.nih.gov/geo/query/acc.cgi?acc=GSE147363). The Uniprot Homo sapiens proteome database (UP000005640) can be accessed at https://www.uniprot.org/proteomes/

UP000005640. The human mature miRNA sequences can be accessed at http://www.mirbase.org/. Source data are provided with this paper.

Field-specific reporting

Please select the one below that is the best fit for your research. If you are not sure, read the appropriate sections before making your selection.

- [x] Life sciences
- [ ] Behavioural & social sciences
- [ ] Ecological, evolutionary & environmental sciences

For a reference copy of the document with all sections, see nature.com/documents/nr-reporting-summary-flat.pdf

Life sciences study design

All studies must disclose on these points even when the disclosure is negative.

Sample size	No statistical methods were used to predetermine sample size. The sample size was chosen to include at least 3 biologically independent experiments (and most often at least 5 biologically independent experiments). Sample size was based on standard sample sizes from our past experiments and similarly to what is described for similar experiments in published articles (cf. Maudet et al. Nat. Commun. 2014 5:4718; Aguilar & Cruz et al. Nat Microbiol. 2020 5:192)
Data exclusions	No data were excluded from the analysis.
Replication	Experiments were independently performed at least 3 times (most often at least 5 biologically independent experiments), and all attempts of replication were successful.
Randomization	Animals were randomly assigned to experimental groups. For in vitro experiments, cultured cells were uniformly plated, with random allocation of treatment (transfection, infection).
Blinding	For miRNA expression analysis, the researchers performing library preparation and next-generation sequencing were blinded to treatment procedures. For mass-spectrometry analysis, the researchers performing sample preparation and analysis were were blinded to treatment procedures. For the remaining experiments, blinding was not applicable given the nature of the reagents/treament used (chemicals, siRNAs, plasmids, and infection). All critical experiments were repeated independently by at least two researchers.

Reporting for specific materials, systems and methods

We require information from authors about some types of materials, experimental systems and methods used in many studies. Here, indicate whether each material, system or method listed is relevant to your study. If you are not sure if a list item applies to your research, read the appropriate section before selecting a response.

Materials & experimental systems

n/a	Involved in the study	
[ ]	[x]	Antibodies
[ ]	[x]	Eukaryotic cell lines
[x]	[ ]	Palaeontology and archaeology
[ ]	[x]	Animals and other organisms
[x]	[ ]	Human research participants
[x]	[ ]	Clinical data
[x]	[ ]	Dual use research of concern

Methods

n/a	Involved in the study	
[x]	[ ]	ChIP-seq
[x]	[ ]	Flow cytometry
[x]	[ ]	MRI-based neuroimaging

Antibodies

Antibodies used	β-actin (1:5,000; Sigma, A2228, RRID:AB_476697), α-tubulin (1:3,000; Sigma, T6074, RRID:AB_477582), E2F1 (1:100; Santacruz, sc-251, RRID:AB_627476), E2F1 (1:1,000; Sigma, SAB2103144, RRID:AB_10666369), IRE1 (1:1,000; Cell Signaling, 3294, RRID:AB_823545), phosphorylated IRE1 (1:1,000; Abcam, ab48187, RRID:AB_873899), BiP (1:1,000; Cell Signaling, 3177, RRID:AB_2119845), HMGB1 (1:100; Santacruz, sc-56698, RRID:AB_783817), ASNS (1:100; Santacruz, sc-365809, RRID:AB_10843357), RAGE (1:100; Santacruz, sc-80652, RRID:AB_1128924), PERK (1:500, Santacruz, sc-377400, RRID:AB_2762850), phosphorylated PERK (1:1,000; Cell Signaling, 3179, RRID:AB_2095853), ATF6 (1:1,000; Abcam, ab122897, RRID:AB_10899171), RTCB (1:1000; proteintech, 19809-1-AP, RRID:AB_10598327), phosphorylated JNK (1:500; Cell Signaling, 4668, RRID:AB_823588), JNK (1:1,000; Cell Signaling, 9252, RRID:AB_2250373), GADPH (1:500; GenScript, A01622-40, RRID:AB_2622160) and anti-mouse and anti-rabbit secondary antibodies coupled to horseradish peroxidase (1:10,000; GE Healthcare, NA931 and NA934, respectively).
Validation	We used commercial antibody reagents for Western-blot and immunofluorescence. The Reseach Resource Identifiers (RRID) for all primary antibodies used in this study are provided in 'Materials and Methods' and section above (Antibodies used). Relevant publications and/or validation results for each antibody can be found through the RRID Portal (https://scicrunch.org/resources), or on the manufacturer's website.<br><br>In addition, the anti-E2F1, anti-IRE1, anti-ATF6, anti-PERK, anti-HMGB1 and anti-RAGE antibodies were validated in our study by

	Western-blot in knockdown samples.

Eukaryotic cell lines

Policy information about cell lines

Cell line source(s)	HeLa-229 (ATCC CCL-2.1) and HCT-8 (ATCC CCL-244) were obtained from the American Type Culture Collection (ATCC).
Authentication	None of the cell lines was authenticated.
Mycoplasma contamination	All cells were tested and negative for mycoplasma contamination.
Commonly misidentified lines (See ICLAC register)	No commonly misidentified cell lines were used in this study.

Animals and other organisms

Policy information about studies involving animals; ARRIVE guidelines recommended for reporting animal research

Laboratory animals	Nine male and female crossbred weaned piglets, of approximately 4 weeks old, were used in this study; all piglets were serologically negative before the infection.
Wild animals	The study did not involve wild animals
Field-collected samples	The study did not involve field collected samples
Ethics oversight	All procedures involving animals were approved by the institutional bioethical committee of the University of Leon, Spain (license number ULE_003_2005, approval date January 25th, 2005) and performed according to European regulations regarding animal welfare and protection of animals used for experimental and other scientific purposes.

Note that full information on the approval of the study protocol must also be provided in the manuscript.
