# Supplementary material for: Reprogramming of microRNA expression via E2F1 downregulation promotes Salmonella infection both in infected and bystander cells
Source: Nat Commun. 2021 Jun 7;12:3392. doi: 10.1038/s41467-021-23593-z (PMC8184997; doi:10.1038/s41467-021-23593-z)
Supplement: Supplementary file 2 — Peer Review File [file 41467_2021_23593_MOESM2_ESM.pdf]

Editorial Note: This manuscript has been previously reviewed at another journal that is not operating a transparent peer review scheme. The manuscript was considered suitable for publication without further review at *Nature Communications*.
